# Characterization of Myeloid Cellular Populations in Mesenteric and Subcutaneous Adipose Tissue of Holstein-Friesian Cows

**DOI:** 10.1038/s41598-020-58678-0

**Published:** 2020-02-04

**Authors:** Bárbara M. Oliveira, Ana Pinto, Alexandra Correia, Paula G. Ferreira, Manuel Vilanova, Luzia Teixeira

**Affiliations:** 1 ICBAS – Instituto de Ciências Biomédicas Abel Salazar, Universidade do Porto, Rua de Jorge Viterbo Ferreira, 228, 4050-313 Porto, Portugal; 2 UMIB – Unidade Multidisciplinar de Investigação Biomédica, Universidade do Porto, Rua de Jorge Viterbo Ferreira, 228, 4050-313 Porto, Portugal; 3 I3S – Instituto de Investigação e Inovação em Saúde, Universidade do Porto, Rua Alfredo Allen, 208, 4200-135 Porto, Portugal; 4 IBMC – Instituto de Biologia Molecular e Celular, Rua Alfredo Allen, 208, 4200-135 Porto, Portugal

**Keywords:** Mast cells, Neutrophils, Eosinophils, Infection, Monocytes and macrophages

## Abstract

Immune cells resident in adipose tissue have important functions in local and systemic metabolic homeostasis. Nevertheless, these immune cell populations remain poorly characterized in bovines. Recently, we described diverse lymphocyte subpopulations in adipose tissue of Holstein-Friesian cows. Here, we aimed at characterising myeloid cell populations present in bovine adipose tissue using multicolour flow cytometry, cell sorting and histochemistry/immunohistochemistry. Macrophages, CD14^+^CD11b^+^MHC-II^+^CD45^+^ cells, were identified in mesenteric and subcutaneous adipose tissue, though at higher proportions in the latter. Mast cells, identified as SSC-A^high^CD11b^−/+^CD14^−^MHC-II^−^CH138A^−^CD45^+^ cells, were also observed in adipose tissue and found at higher proportions than macrophages in mesenteric adipose tissue. Neutrophils, presenting a CH138A^+^CD11b^+^ phenotype, were also detected in mesenteric and subcutaneous adipose tissue, however, at much lower frequencies than in the blood. Our gating strategy allowed identification of eosinophils in blood but not in adipose tissue although being detected by morphological analysis at low frequencies in some animals. A population not expressing CD45 and with the CH138A^+^ CD11b^−^MHC-II^−^ phenotype, was found abundant and present at higher proportions in mesenteric than subcutaneous adipose tissue. The work reported here may be useful for further studies addressing the function of the described cells.

## Introduction

Studies in humans and mice have been showing the importance of immune cells resident in adipose tissue to the local and systemic metabolic homeostasis^1,2^. Macrophages are one of the populations best studied due to their involvement in metabolic diseases resulting from human obesity^1^, but other leukocyte populations present in the adipose tissue have been involved in diverse physiological functions. Murine eosinophils are important for the maintenance of alternatively activated macrophages in the adipose tissue^3^ and for cold-induced thermogenic beige fat^4^ while functional inactivation of mast cells increases adipocyte browning and thermogenesis^5^. Neutrophils are also present in mouse and human adipose tissue^1,6^ and may contribute to associated inflammation in obesity^7,8^. Moreover, lymphocytes have an important role in maintaining homeostasis of the adipose tissue^2,9^. On the other hand the adipose tissue can also have important contributions for the immune function^8,10^, as for example being a reservoir for memory T cells with potential to protect from infection^11,12^. The adipose tissue has also gained major interest as a source of non-immune cells, such as stem cells due to their potential in human^13–15^ and veterinary medicine^16,17^. Studies in mice and humans have clearly shown the complexity of cell populations that exist in adipose tissue^7,18,19^ and much has to be done to elucidate their functional interactions^1,8^. Despite this, cell populations in bovine adipose tissue remain poorly characterized. In a previous work we have shown the presence of CD8^+^ and CD4^+^ T cells, γδ T cells, as well as NK cells^20^. In this work, we aimed to characterize myeloid cell populations in bovine adipose tissue. In bovines, identification of adipose tissue macrophages has relied on using one^21–24^ or two markers simultaneously^25^. By immunohistochemistry CD68^+^ cells, CD11b^+^ cells, CD14^+^ cells and CD11c^+^ cells were previously shown to be present in bovine adipose tissue^21,22^. Single colour-flow cytometry revealed the presence of CD14^+^ cells, CD172a^+^ cells, CD11c^+^ cells and CD163^+^ cells in the stromal vascular fraction (SVF) of subcutaneous and omental adipose tissue^23,24^. Recently, Aylward *et al*.^25^ reported the presence of CD172a^+^CD11b^+^cells, CD11c^+^MHC-II^+^ cells, and CD11b^+^CD11c^+^ cells in mesenteric adipose tissue. However, these markers are not exclusive to macrophages but common to other myeloid cell populations or can be even expressed in non-hematopoietic cells. As an example, studies in humans and mice have shown that CD14 can be expressed in non-myeloid cells^26^ such as preadipocytes^27^ and epithelial cells^28^. Also, CD172a is expressed by granulocytes such as eosinophils^29^ and mast cells^30^ beside its expression in monocytes/macrophages. Since mast cells^31,32^ and eosinophils^3,33^ have already been described in adipose tissue, single markers analysis limits the interpretation of the data. Therefore, to characterize the different myeloid cell populations in bovine adipose tissue we used a multicolour flow cytometry panel associated with cell sorting and histochemistry/immunohistochemistry analysis. Our strategy allowed identification and isolation of macrophages, mast cells and neutrophils with macrophages being observed at different proportions in mesenteric adipose tissue (MAT) and subcutaneous adipose tissue (SAT). We also showed that CD45 negative cells represent more than 40% of all the cells in the adipose tissue SVF of MAT. A non-leukocyte population was also observed at different proportions in MAT and SAT, although its identity remains to be determined in future studies. Overall, our results highlight that distinct adipose tissue depots may present distinct cellular characteristics.

## Results

### Identification of macrophages in bovine adipose tissue

**Figure 1:**
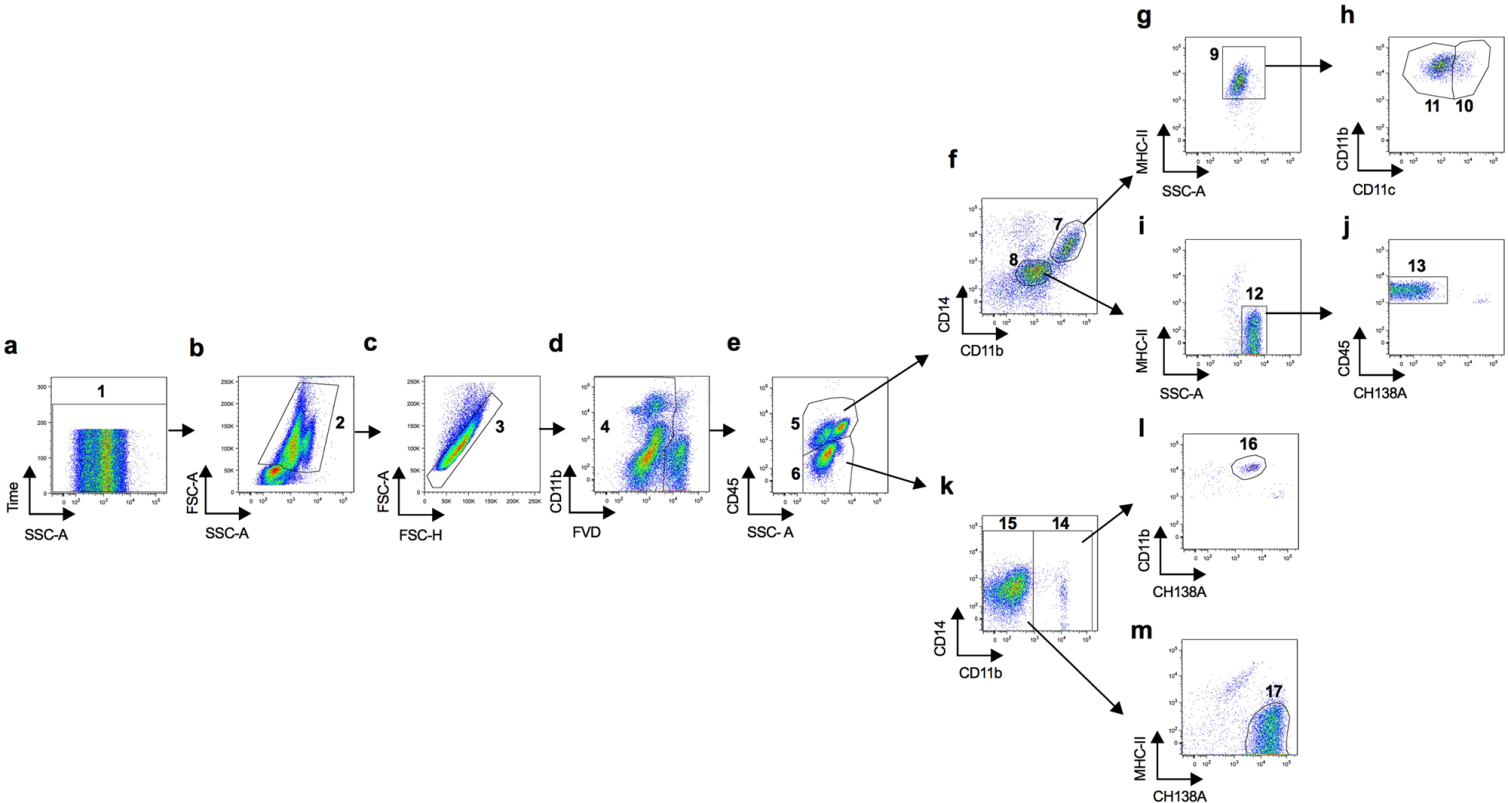
Flow cytometry gating strategy used to define cell populations from the stromal vascular fraction of bovine subcutaneous adipose tissue. (**a**) Time parameter allowed exclusion of events bursts (1). (**b**) Selection of cells without debris (2) and (**c**) singlets (3). **(d**) Dead cells exclusion with Fixable Viability Dye (FVD) (4). (**e**) Selection of CD45^+^ (5) and CD45^−^ (6) cells. (**f**) Selection of CD14^+^CD11b^+^(7) or CD11b^−/+^CD14^−^ cells (8). (**g**) Gate used to analyse CD14^+^CD11b^+^MHC-II^+^CD45^+^ cells (9) (macrophages). (**h**) Gate used to identify CD11c^+^ (10) and CD11c^−^ (11) macrophages. (**i**) Selection of SSC-A^high^ MHC-II^−^ cells (12). (**j**) Gate used to analyse SSC-A^high^CD11b^−/+^CD14^−^MHC-II^−^CH138A^−^CD45^+^ cells (13) (mast cells). (**k**) Selection of CD11b^+^ (14) or CD11b^−^ (15) cells in CD45^−^ cells. (**l**) Gate used to analyse CH138A^+^CD11b^+^ cells (16) (neutrophils). (**m**) Gate used to analyse CH138A^+^CD11b^−^MHC-II^−^CD45^−^ cells (17). Pseudocolor plots are representative examples of the analysis of SVF cells isolated from SAT.

**Figure 2:**
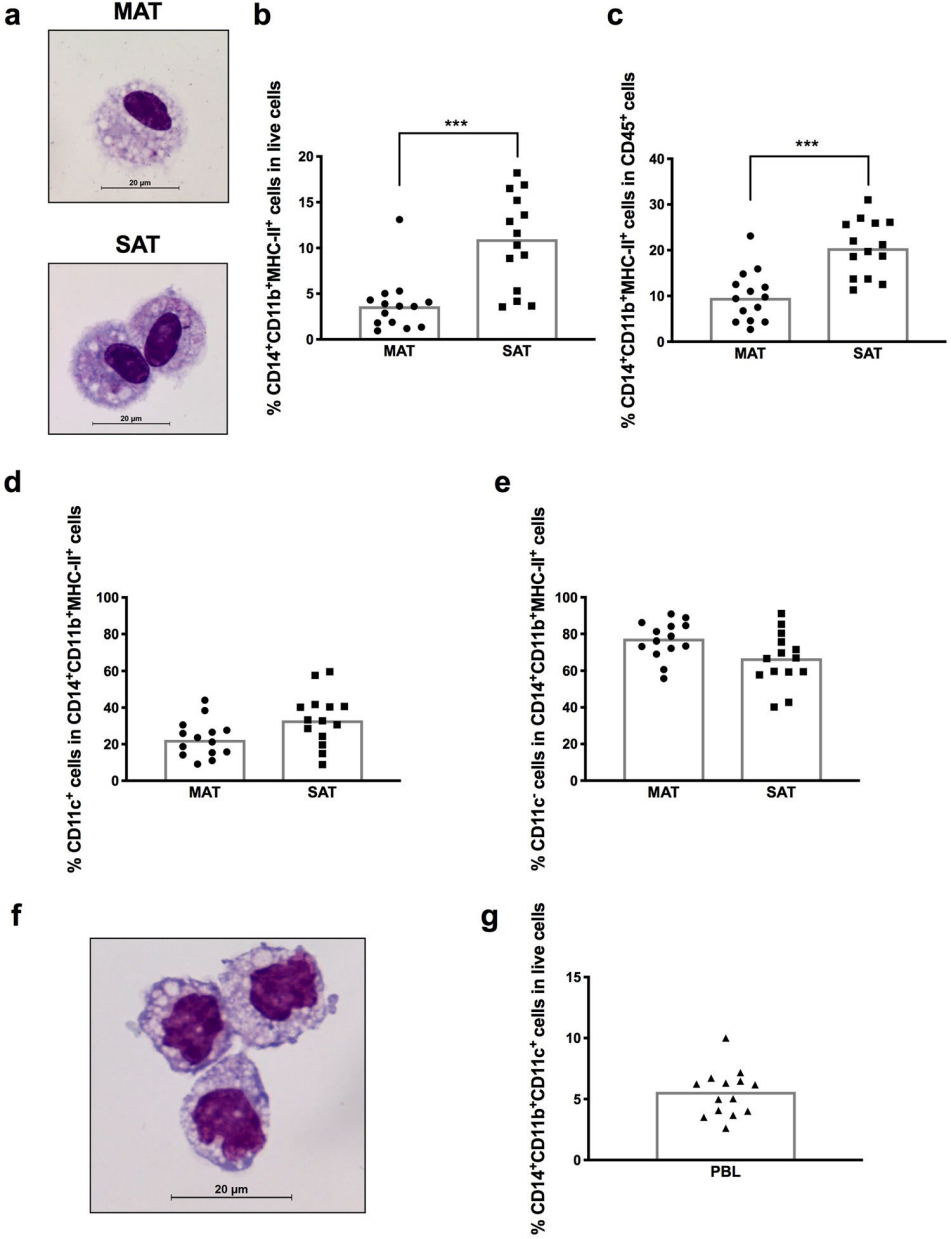
Macrophages in adipose tissue. (**a**) Representative May-Grünwald-Giemsa staining of sorted CD14^+^CD11b^+^MHC-II^+^CD45^+^ cells is shown for mesenteric and subcutaneous bovine adipose tissue (MAT and SAT, respectively). Bar = 20 μm. Frequencies of CD14^+^CD11b^+^MHC-II^+^CD45^+^ cells in (**b**) total live stromal vascular fraction cells and (**c**) CD45^+ ^cells isolated from MAT and SAT. Frequencies of (**d**) CD11c^+^ and (**e**) CD11c^−^ cells on total CD14^+^CD11b^+^MHC-II^+^CD45^+^ cells in MAT and SAT. (**f**) Representative May-Grünwald-Giemsa staining of sorted CD14^+^CD11b^+^CD11c^+^ cells (monocytes) is shown. Bar = 20 μm. (**g**) Frequencies of CD14^+^CD11b^+^CD11c^+^ cells in leukocytes isolated from whole blood (PBL). In graphics, each symbol represents an individual animal. Bars represent medians of 14 bovines per group pooled from 5 independent experiments. Statistically significant differences between different tissues are indicated. (Wilcoxon matched-pairs signed rank test; ***P < 0.001).

**Figure 3:**
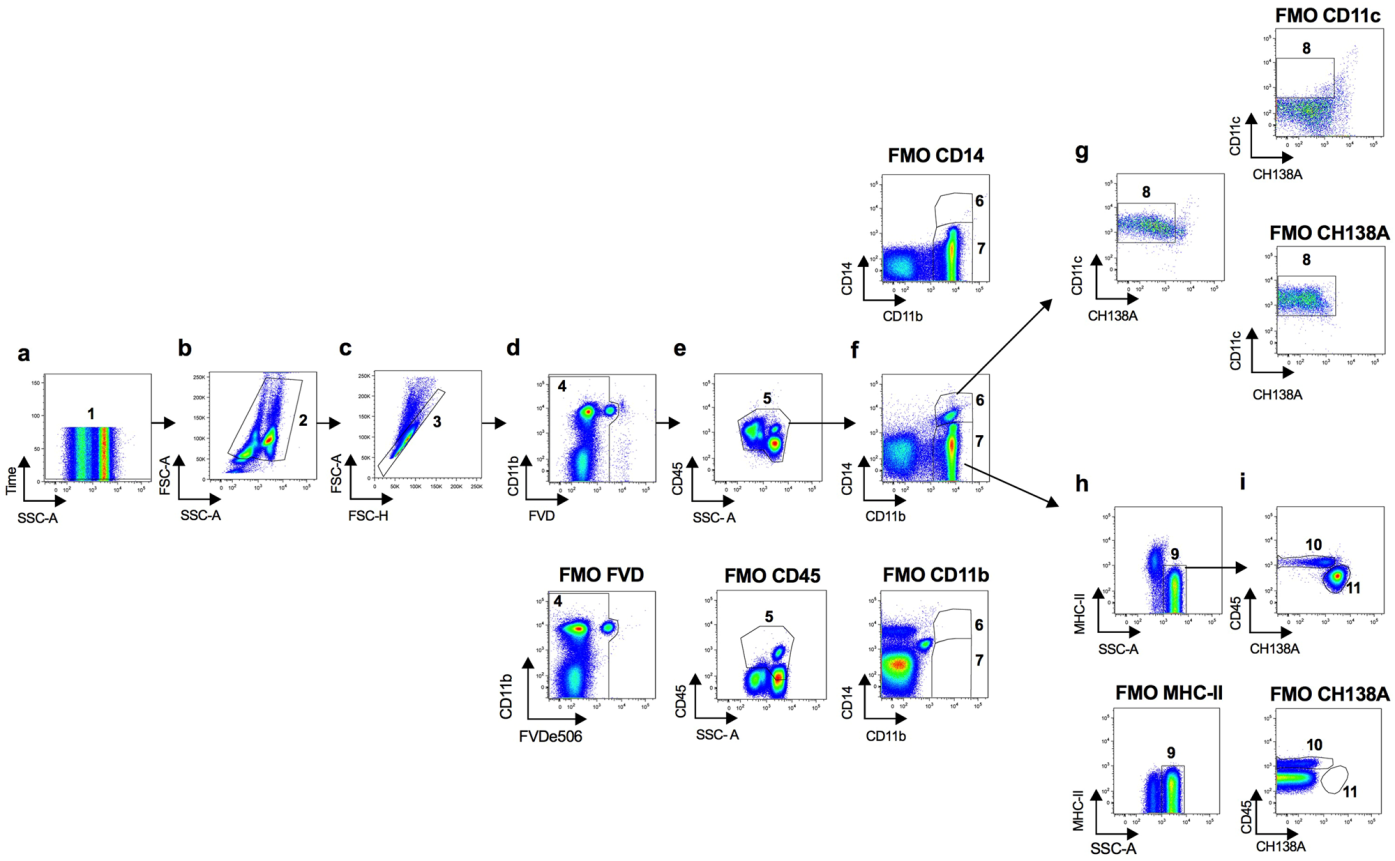
Flow cytometry gating strategy used to define cell populations from bovine peripheral blood. (**a**) Time parameter allowed exclusion of events bursts (1). (**b**) Selection of cells without debris (2) and (**c**) singlets (3). (**d**) Dead cells exclusion with Fixable Viability Dye (FVD) (4). (**e**) Selection of CD45^+^ cells (5). (**f**) Selection of CD14^+^CD11b^+^ (6) or CD11b^+^CD14^−^ cells (7). (**g**) Gate used to analyse CD14^+^CD11b^+^CD11c^+^ cells (8) (monocytes). (**h**) Selection of SSC-A^high^MHC-II^−^ cells (9). (**i**) Gate used to analyse SSC-A^high^CD11b^+^CD14^−^MHC-II^−^CH138A^−/int^ cells (10) (eosinophils) and CH138A^+^CD11b^+^SSC-A^high^ cells (11) (neutrophils). Respective Fluorescence Minus One (FMO) controls are presented, as indicated.

Macrophages were selected from the CD45^+^ population as cells simultaneously expressing CD14, CD11b and MHC-II (Fig. 1, Supplementary Figs. S1 and S2 for gating controls). CD45 marker was used to identify leukocytes. We included in our panel CD14 and CD11b as these markers are commonly used to define macrophages in adipose tissue of humans^34,35^. We also included MHC class II (MHC-II) as macrophages in murine adipose tissue have been described as displaying a MHC-II^high^ phenotype^36^. The suitability of our choice of markers was assessed by cell sorting using the full gating strategy shown in Supplementary Fig. S3. Sorted CD14^+^CD11b^+^MHC-II^+^CD45^+^ cells presented macrophage morphology (Fig. 2a and Supplementary Fig. S3), validating our approach. The observed frequency of macrophages in MAT was 3,62% (median value) of total live SVF cells (range 0,937 to 13,1%) and 9,57% of all CD45^+^ cells (range 2,67% to 23,1%) (Fig. 2b,c). In SAT a higher frequency of macrophages was observed comparatively to MAT (median value of 10,95% and 20,45% in total SVF cells and CD45^+^ cells, respectively), ranging from 3,55 to 18,2% of total SVF cells and 11,33 to 31% of all CD45^+^ cells (Fig. 2b,c). Indeed, in all animals analysed but one, the frequency of macrophages was higher in SAT than MAT (Supplementary Figs. S4 and S5). Monocytes in bovine blood were previously described as cells expressing CD14, CD11c and CD11b^37,38^. Accordingly, CD14^+^CD11b^+^CD11c^+^ cells sorted from PBL presented monocyte morphology (Fig. 2f and Supplementary Fig. S6). The frequency of monocytes in the analysed animals was 5,6% (median value) of all PBL (Fig. 2g and Fig. 3 for gating strategy). Only monocytes positive for CD14 were selected. Therefore nonclassical monocytes, that do not express CD14^39^, were not analysed. In contrast to blood monocytes, CD11c expression was only observed in a fraction of adipose tissue macrophages (22,4% and 33,05% of total macrophages in MAT and SAT, respectively) (Fig. 2d). No differences in the frequency of CD11c^+^ or CD11c^−^ macrophages were observed between MAT and SAT (Fig. 2d,e).

### Identification of granulocytes in bovine adipose tissue

**Figure 4:**
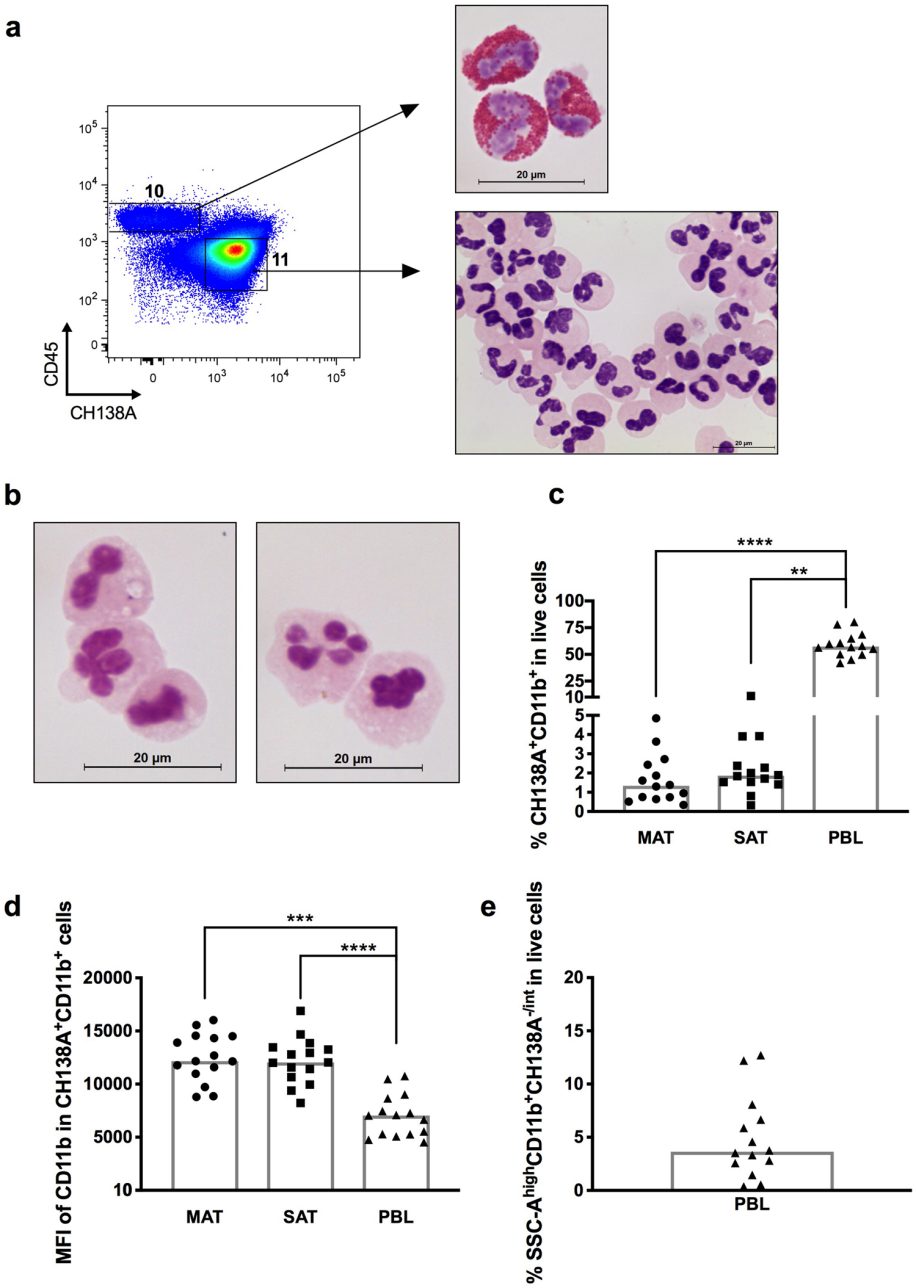
Polymorphonucleares granulocytes in adipose tissue. (**a**) Representative example of gating strategy used to cell-sort SSC-A^high^CD11b^+^CD14^−^MHC-II^−^CH138A^−/int^ cells (10) (eosinophils) and CH138A^+^CD11b^+^SSC-A^high^ cells (11) (neutrophils) from peripheral blood leukocytes (PBL) and respective May-Grünwald-Giemsa staining. Bar = 20 μm. (**b**) Representative May-Grünwald-Giemsa staining of CH138A^+^CD11b^+^ cells (neutrophils) isolated from subcutaneous adipose tissue (SAT). (**c**) Frequencies of CH138A^+^CD11b^+^ cells (neutrophils) in total SVF cells isolated from mesenteric adipose tissue (MAT), SAT and in PBL. (**d**) Median fluorescence intensity (MFI) values of CD11b in CH138A^+^CD11b^+^ cells in MAT, SAT and PBL, as indicated. (**e**) Frequency of SSC-A^high^CD11b^+^CD14^−^MHC-II^−^CH138A^−/int^ cells (eosinophils) in leukocytes isolated from whole blood. Each symbol represents an individual animal. Bars represent medians of 14 bovines per group pooled from 5 independent experiments. Statistically significant differences between different tissues are indicated. (Friedman test with Dunn’s multiple comparisons test; **P < 0.01; ***P < 0.001; ****P < 0.0001).

The CH138A mAb was reported to bind granulocytes in bovine peripheral blood^40^ and milk neutrophils^41^. Here, we observed by immunocytochemistry analysis that this antibody binds to polymorphonuclear cells in PBL, although not all (Supplementary Fig. S7). Using this antibody and cell sorting we were able to identify and separate bovine blood neutrophils (Fig. 4a and Supplementary Fig. S6). Others have reported that CD45 expression together with side scatter (SSC-A) parameter, indicative of cell granularity, could be used to differentiate neutrophils from monocytes and lymphocytes in bovine blood^42^. Similarly, we observed that some neutrophils in PBL have low expression of CD45. Indeed, if we considered the respective FMO to define the gate for CD45^+^ cells in PBL, the neutrophil population would be cut in half (Fig. 3e). Accordingly, by immunocytochemistry we observed that some polymorphonuclear cells in PBL have lower brown coloration than surrounding cells, indicating lower expression of CD45 at cell surface (Supplementary Fig. S7). Therefore, in PBL we considered all the population to be CD45 positive, regardless of the FMO control (Fig. 3e). In accordance with the observation on PBL, adipose tissue neutrophils were found within the CD45^−^ region and presented a CD11b^+^CH138A^+^ phenotype as confirmed by cell sorting (Figs. 1l and 4b, Supplementary Fig. S3). The observed frequency of this cellular population in bovine adipose tissue was low representing only 1,34% and 1,86% of total cells in MAT and SAT, respectively (Fig. 4c). No differences in the frequency were found between MAT and SAT. In contrast, neutrophils, the most abundant population in PBL (Supplementary Fig. S8), accounted for 57,45% of all PBL and their frequency was higher than in adipose tissue (Fig. 4c). CD11b expression on PBL neutrophils was lower than on MAT and SAT neutrophils (Fig. 4d).

To the best of our knowledge there is no single marker allowing identification of eosinophils in bovines. Nevertheless, eosinophils in blood can be sorted relying in high SSC-A and high autofluorescence^43,44^. Accordingly, we sorted a PBL population with high SCC-A and high autofluorescence in the 488 nm region and verified that it consisted of eosinophils with their characteristic bilobed nucleus and the marked acidophilic granules (coloration brick-red) upon the May-Grünwald-Giemsa stain (Supplementary Fig. S6). In half of the analysed animals (n = 7 out of 14 in flow cytometry analysis and n = 2 out of 4 in cell sorting) an intermediary level of fluorescence for CH138A was detected in eosinophils, showing that CH138A can also bind to eosinophils in some animals (Supplementary Fig. S6). Only the higher signal in the CD45 channel (due to higher autofluorescence and/or higher CD45 expression) allowed their separation from neutrophils as illustrated by the example shown in Figs. 3i and 4a. Moreover, blood eosinophils stained CD11b positive but negative to CD14 and MHC-II (Fig. 3f,h,i, Supplementary Fig. S6), and accounted for 3,64% of all PBL (Fig. 4e).

**Figure 5:**
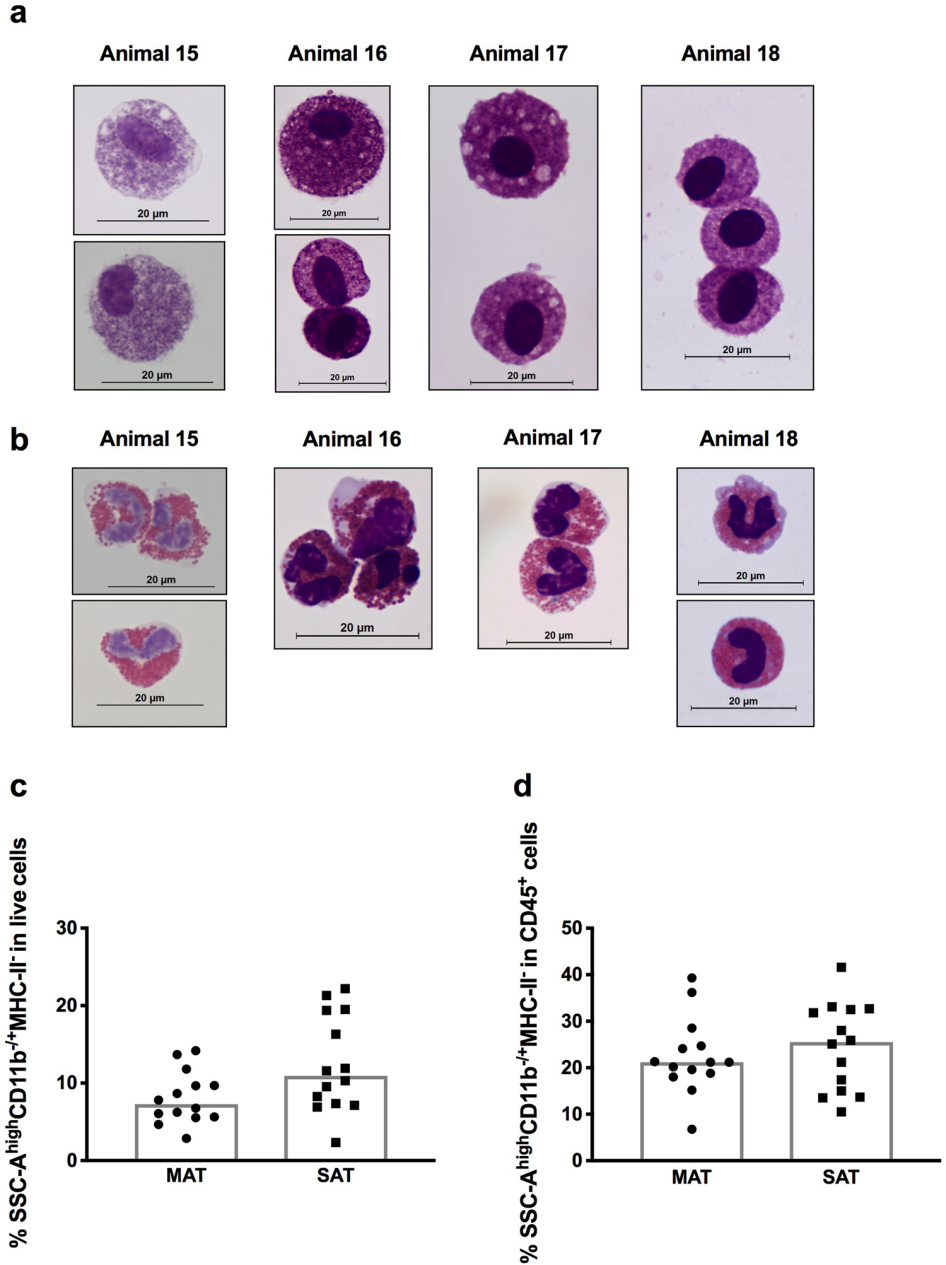
Granulocytes non-polymorphonuclear in adipose tissue. Representative May-Grünwald-Giemsa staining of (**a**) sorted SSC-A^high^CD11b^−/+^CD14^−^MHC-II^−^CH138A^−^CD45^+^ cells (mast cells) from subcutaneous adipose tissue (SAT) and (**b**) corresponding eosinophils in blood, from 4 independent experiments are shown. Bar = 20 μm. Frequencies of SSC-A^high^CD11b^−/+^CD14^−^MHC-II^−^CH138A^−^CD45^+^ cells (mast cells) in (**c**) total live stromal vascular fraction cells and (**d**) CD45^+^ cells isolated from mesenteric bovine adipose tissue (MAT) and SAT. Each symbol represents an individual animal. Bars represent medians of 14 bovines per group pooled from 5 independent experiments. No statistically significant differences between different tissues were found (Wilcoxon matched-pairs signed rank test).

In adipose tissue, we also identified a SSC-A^high^CD14^−^MHC-II^−^CH138A^−^CD45^+^ population with high autofluorescence. However, in this cell population some cells stained negative and some slightly positive for CD11b (CD11b^−/+^) contrastingly to blood eosinophils that stained CD11b^high^ (Fig. 1f–j and Supplementary Fig. S3). After cell sorting and upon May-Grünwald-Giemsa staining, we verified that cells in this cell population were heterogeneous in size, with round or oval nucleus contrarily to PBL eosinophils that presented a bilobed nucleus (Fig. 5a,b). Moreover, the granules observed in the cytoplasm were purple and not brick-red as the ones observed in the cytoplasm of PBL eosinophils of the respective animals (Fig. 5a,b). The morphology and staining we observed is therefore consistent with that of mast cells that upon May-Grünwald-Giemsa stain are described as oval or angular cells with purple granules and blue nucleus^45–47^. Tryptases are enzymes quite abundant in mast cells and used to identify this cellular population^48–50^. Therefore, for additional confirmation that this population was indeed mast cells, we also assess the expression of tryptase beta 2 gene (*TPSB2*) in the sorted SSC-A^high^CD11b^−/+^CD14^−^MHC-II^−^CH138A^−^CD45^+^ population. Expression of *TPSB2* was detected in all samples tested (SSC-A^high^CD11b^−/+^CD14^−^MHC-II^−^CH138A^−^CD45^+^ cells sorted from three samples of SAT and MAT) (Supplementary Fig. S9). Contrastingly, no expression was detected in PBL (Supplementary Fig. S9), consistent with the fact that mast cells are resident in tissues and not found in the blood under normal conditions^51^. Although β-tryptases^50^ can also be expressed by basophils, no cells with segmented nucleus, characteristic of basophils^52^, were observed. Therefore, our results show that SSC-A^high^CD11b^−/+^CD14^−^MHC-II^−^CH138A^−^CD45^+^ cells are indeed mast cells. This population accounted for 7,29% and 10,95% of all SVF cells in MAT and SAT, respectively (Fig. 5c). In CD45^+^ cells, this populations accounted for 21,2% and 25,55% in MAT and SAT, respectively (Fig. 5d). In MAT the frequency of this cell population was found higher than the one of macrophages (Figs. 2 and 5, p = 0,0006; Wilcoxon matched-pairs signed rank test). Indeed, in all analysed animals but one, the frequency of mast cells was higher than the one of macrophages (Animal 6 of Supplementary Fig. S4). No difference was found in the proportions of mast cells between MAT and SAT (Fig. 5c,d). Contrastingly to mast cells, we were not able to identify eosinophils in adipose tissue using our flow cytometry strategy. Nevertheless, eosinophils were rarely observed in SAT and MAT SVF upon morphological analysis of cytospin preparations (Supplementary Fig. S10). In SAT the median frequency of this population determined by morphological analysis was only 0,66% of total SVF cells and undetected in 2 out of 7 animals (Supplementary Fig. S10). The frequency, of eosinophils was significantly lower than the frequency of mast cells upon morphological analysis (Supplementary Fig. S10). This may have contributed to the difficulty of identifying this cell population using flow cytometry.

### CD45 negative cells in bovine adipose tissue

**Figure 6:**
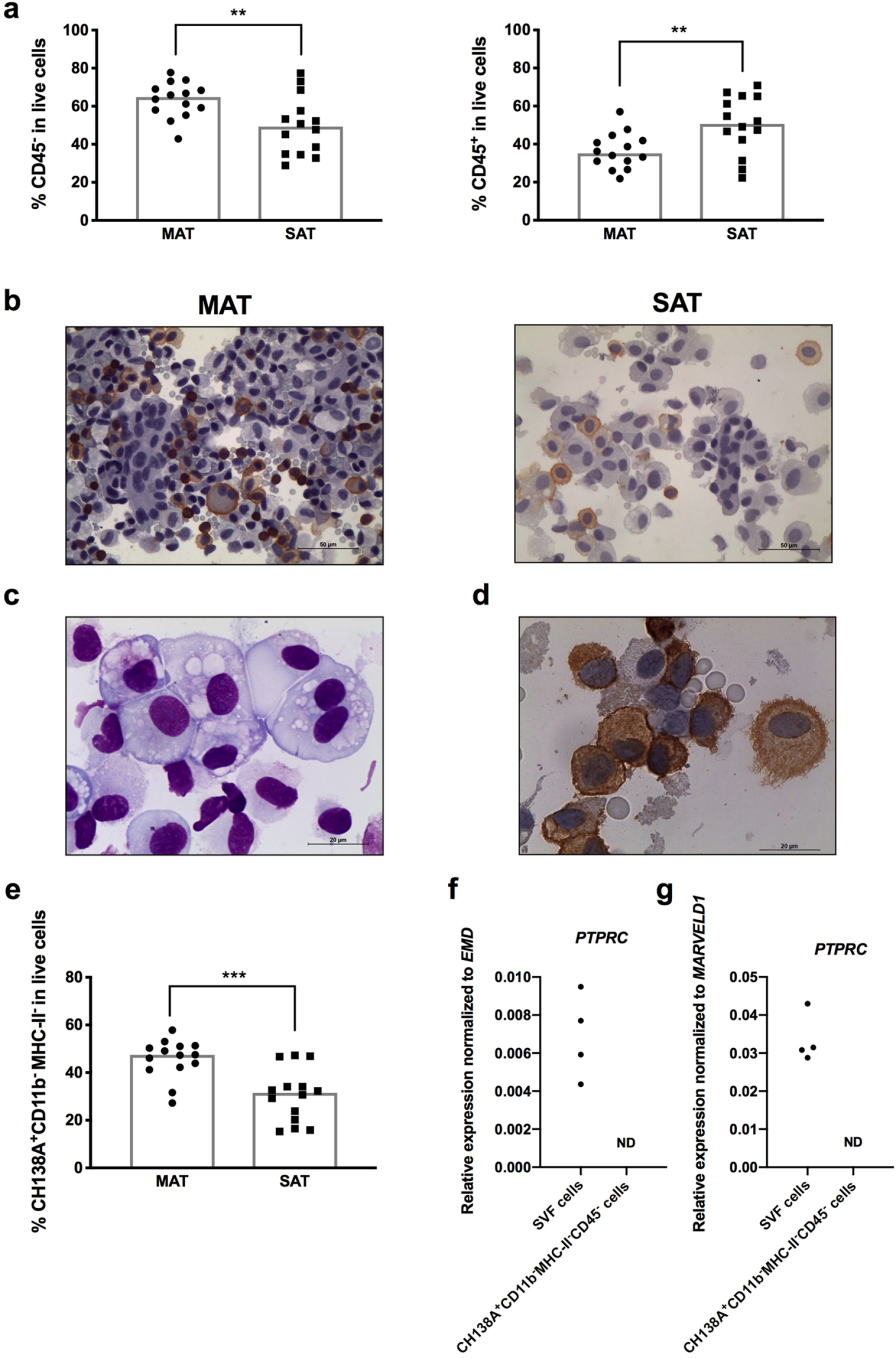
CD45 negative cells. (**a**) Frequencies of CD45^−^ and CD45^+^ cells in total stromal vascular fraction cells (SVF) cells isolated from mesenteric and subcutaneous bovine adipose tissue (MAT and SAT, respectively). (**b**) Representative immunocytochemistry analysis of CD45 in SVF cells isolated from MAT and SAT. Cells were specifically stained (brown coloration) with a monoclonal mouse anti-bovine CD45 and counterstained with haematoxylin. Bar = 50 μm. (**c**) Representative May-Grünwald-Giemsa of sorted CH138A^+^CD11b^−^MHC-II^−^CD45^−^ cells. Bar = 20 μm. (**d**) SVF cells isolated from MAT were stained (brown coloration) with the monoclonal antibody clone CH138A and counterstained with haematoxylin. Bar = 50 μm. (**e**) Frequencies of CH138A^+^CD11b^−^MHC-II^−^CD45^−^ cells in total SVF cells isolated from MAT and SAT. In graphics, each symbol represents an individual animal. Bars represent medians of 14 bovines per group pooled from 5 independent experiments. Statistically significant differences between different tissues are indicated. (Wilcoxon matched-pairs signed rank test; **P < 0.01, ***P < 0.001). Relative levels of protein tyrosine phosphatase receptor type C (*PTPRC*) mRNA normalized to (**f**) emerin (*EMD*) and g) MARVEL domain containing 1 (*MARVELD1*) mRNA as indicated, determined by real-time PCR in SVF cells isolated from SAT or in sorted CH138A^+^CD11b^−^MHC-II^−^CD45^−^ cells isolated from the SVF of SAT. Each symbol represents an individual animal (n = 4 for SAT SVF cells and sorted CH138A^+^CD11b^−^MHC-II^−^CD45^−^ cells, ND = not detected).

Flow cytometry analysis clearly showed that in bovine adipose tissue there is a high frequency of CD45 negative cells, higher in MAT than SAT, that in some animals can represent the majority of SVF cells (Fig. 6a). The frequency of CD45 negative cells in total live cells ranged from 42,9–77,7% in MAT and 28,9–77,4% in SAT. Immunocytochemistry analysis of CD45 on total SVF cells showed the presence of many cells that did not show expression of CD45 (Fig. 6b). By flow cytometry analysis, a population of CD45^−^ cells staining positive for CH138A mAb and negative for CD11b and MHC-II was clearly observed in SAT and MAT (Fig. 1e,k,m and Supplementary Fig. S1, respectively). Upon cell sorting we verified that many of these cells have macrophage-like morphology and do not present the granulocytic morphology typical of neutrophils (Fig. 6c and Supplementary Fig. S3). Immunocytochemistry analysis of SVF cells of adipose tissue also revealed the presence of many non-polymorphonuclear cells with macrophage-like morphology staining with the CH138A mAb (Fig. 6d and Supplementary Fig. S7). This CH138A^+^CD11b^−^MHC-II^−^CD45^−^ population is quite abundant, accounting for 47,4% and 31,5% of all SVF cells in MAT and SAT, respectively, being significantly higher in MAT than SAT (Fig. 6e). Indeed, in MAT, this cell population was the one with highest frequency in all animals analysed, except one in which the frequency of all other CD45^+^ cells (includes all CD45^+^ cells except the macrophages and mast cells) was higher (Supplementary Fig. S5). The frequency of this cell population was higher than that of macrophages in both MAT and SAT (p = 0,0001 and p = 0,0017, for MAT and SAT, respectively; Wilcoxon matched-pairs signed rank test) (Figs. 2b and 6e). For additional confirmation that CH138A^+^CD11b^−^MHC-II^−^CD45^−^ cells were indeed CD45 negative, we also assessed the expression of the gene encoding this molecule, protein tyrosine phosphatase receptor type C gene (*PTPRC*) in the sorted populations. No *PTPRC* expression was detected in sorted CH138A^+^CD11b^−^MHC-II^−^CD45^−^ cells (Fig. 6f,g), although expression was detected for the constitutive genes emerin (*EMD*) and MARVEL domain containing 1 (*MARVELD1*) (data not shown). As a control, *PTPRC* expression was assessed in total SVF cells and, as expected, CD45 expression was detected in all samples analysed (Fig. 6f,g).

### Comparative analysis of myeloid cell populations in the adipose tissue of animals seropositive or seronegative for *Neospora caninum*

**Figure 7:**
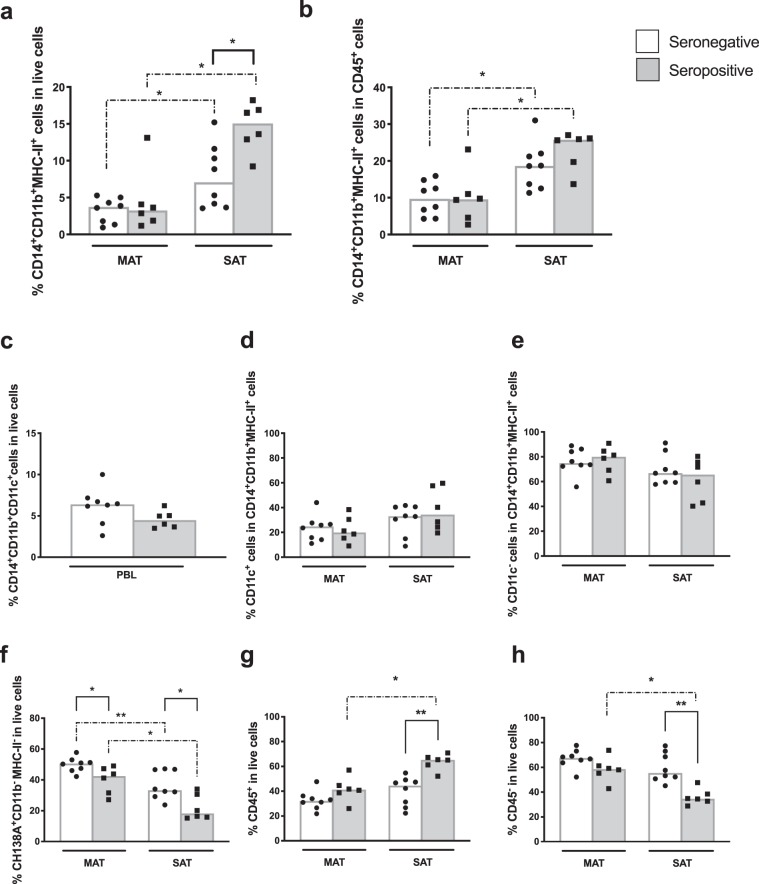
Macrophage and CH138A^+^CD11b^−^MHC-II^−^CD45^−^ cells in *N. caninum* seropositive animals. Frequencies of CD14^+^CD11b^+^MHC-II^+^CD45^+^cells (macrophages) in (**a**) total live stromal vascular fraction cells (SVF) and (**b)** CD45^+^ cells isolated from mesenteric and subcutaneous bovine adipose tissue (MAT and SAT, respectively) from bovines seronegative (white bars) or seropositive (grey bars) to *N. caninum* as indicated. **(c)** Frequencies of CD14^+^CD11b^+^CD11c^+^ cells (monocytes) in leukocytes isolated from whole blood (PBL) from bovines seronegative (white bars) or seropositive (grey bars) to *N. caninum*. Frequencies of (**d)** CD11c^+^ and (**e)** CD11c^−^ cells on total CD14^+^CD11b^+^MHC-II^+^CD45^+^cells in MAT and SAT from bovines seronegative (white bars) or seropositive (grey bars) to *N. caninum*. Frequencies of (**f)** CH138A^+^CD11b^−^MHC-II^−^CD45^−^cells, (**g)** CD45^+^ cells, and (**h)** CD45^−^ cells in total SVF cells isolated from MAT and SAT from bovines seronegative (white bars) or seropositive (grey bars) to *N. caninum*, as indicated. Each symbol represents an individual animal. Bars represent means of 6 or 8 bovines per group pooled from 5 independent experiments. Statistically significant differences between different tissues are indicated. (Mann-Whitney U, *P < 0.05; **P < 0.01).

It has been shown in the murine model that *N. caninum* can impact on the frequencies of immune populations present in adipose tissue^12,53^. Since *N. caninum* infection is highly prevalent in Portuguese bovine herds, where seropositivity to this parasite can reach 80%^54^, we reanalysed our data splitting the animals in seropositive and seronegative to *N. caninum*. Seropositive animals presented an increased frequency of macrophages in SAT total SVF comparatively to seronegative ones (Fig. 7a). No such difference was observed in the frequency of MAT macrophages and PBL monocytes (Fig. 7a–c), as well as of CD11c macrophages (Fig. 7d,e). Contrastingly to macrophages, a decreased frequency of the CH138A^+^CD11b^−^MHC-II^−^CD45^−^ population was observed in MAT and SAT of the animals seropositive to *N. caninum* (Fig. 7f). Overall, seropositive animals presented increased frequencies of CD45^+^ cells and decrease frequencies of CD45^−^ cells when compared to seronegative ones in SAT (Fig. 7g,h). No different frequency of mast cells and neutrophils was found between seronegative and seropositive animals (Supplementary Fig. S11).

## Discussion

Many studies in mice and humans have shown the diversity of cellular populations present in adipose tissue as well as their important contribution to the metabolic host homeostasis^1,2^. Contrastingly, in bovines few studies characterized the cellular populations present in this tissue. In a previous work we showed diverse lymphoid subpopulations present in bovine adipose tissue, namely CD4^+^ and CD8^+^CD3^+^ non-γδ T cells, γδ-T cells and NK cells, with the majority of T cells presenting an effector memory phenotype^20^. Here, we extended these observations by describing the complexity of myeloid cell populations that can be found in this tissue. Macrophages, defined here as CD14^+^CD11b^+^MHC-II^+^CD45^+^ cells, were observed at different proportions in subcutaneous and mesenteric adipose tissue highlighting that adipose tissue of distinct anatomical locations may present distinct features in what regards immune cells. Similarly to what was observed in studies in humans^35^ and mice^55^, a fraction of adipose tissue macrophages were CD11c^+^. In mice, CD11c-expressing macrophages have higher expression of proinflammatory genes compared to CD11c^−^ macrophages^55^. The gating strategy described in this work can be used in future studies to assess if this subtype of macrophages may have similar characteristics in bovines. Contrastingly to work reported by others in bovine omental adipose tissue^56^, we observe that macrophages were not the predominant cell population in bovine MAT and SAT. CH138A^+^CD11b^−^MHC-II^−^CD45^−^ cells were one of the more prevalent populations in bovine adipose tissue. A possible explanation for this apparent discrepancy could be due to the particular adipose tissue depot studied by the authors or due to the fact that macrophage identification in that study was made using anti-mouse acid phosphatase 5 and mac-3/lamp-2 antibodies to identify lysosomes^56^, organelles that are not restricted to macrophages, but present in all eukaryotic cells^57^. Indeed, other authors reported frequencies of CD14^+^ cells (a marker that can be found in macrophages but also on other cells) in total SVF of healthy nonlactating nongestating cows of only 4,42% in omental adipose tissue and 4,08% in subcutaneous adipose tissue^24^.

Similarly to previous observations in humans and mice^58^, neutrophils were found in low frequency in bovine adipose tissue, contrastingly to blood where they represent more than half of the leukocytes. As neutrophils are found in low frequency in bovine adipose tissue and highly represented in the blood it cannot be excluded that these cells may be circulating neutrophils as some erythrocytes were also observed in the isolated SVF. Nevertheless, the expression levels of CD11b in adipose tissue neutrophils are higher than the ones in blood, suggesting that this cellular population is distinct from the one found in blood. Increased levels of CD11b on neutrophils present in nasal mucosa comparatively to the ones in the blood have also been shown in healthy humans^59^. Moreover, CD11b up-regulation has been shown in activated neutrophils^60^. In contrast to neutrophils, eosinophils were found to represent in median less than 5% of all leukocytes in blood. Our gating strategy did not allow eosinophils isolation from the total adipose tissue SVF cells, as from blood, likely due to their low frequency in bovine adipose tissue. Our results are in clear contrast with a recent report describing eosinophils as one of the most abundant population in bovine adipose tissue (57,45% of all stromal vascular fraction cells in SAT)^61^. The reason for this discrepancy may be due to a different morphological criterion used by the authors to identify eosinophils. Our results are nevertheless in line with human studies where a frequency of only 2,34% of total SVF in SAT was reported^62^. Moreover, in lean mice eosinophils account only to 4 to 5% of all SVF cells of perigonadal adipose tissue^3^. Another population of granulocytes that we observed in the bovine adipose tissue was mast cells. Sorted mast cells from bovine adipose tissue presented morphology and phenotype similar to mast cells described in human bone marrow^63^, such as heterogeneous size and granularity, autofluorescence, negative for the CD14 marker, variable CD11b reactivity and tryptase expression. Previous studies have already reported the presence of mast cells in bovine subcutaneous adipose tissue^64^ and far stroma in mammary tissue^45^ by colorimetric staining with astrablau and May-Grünwald-Giemsa, respectively. Tryptase-positive mast cells have also been previous reported in the perivascular area of bovine skin^65^. Here we identified mast cells by flow cytometry as having a SSC-A^high^CD11b^−/+^CD14^−^MHC-II^−^CH138A^−^CD45^+^ profile, being their identity confirmed by colorimetric staining and by expression of the *TPSB2* gene. Although tryptase beta 2 expression levels varied between samples, likely due to the fact that RNA was extracted from a small number of sorted cells, we were nevertheless able to detect the expression of this gene in all the six samples tested. In bovine adipose tissue, mast cells accounted for 21,2% and 25,55% of all CD45^+^ cells in MAT and SAT, respectively, being even more frequent than macrophages in MAT. This contrasts with the murine model, where the percentage in total CD45^+^ cells of mast cells in epididymal adipose tissue is less than 2% while that of macrophages is more than 20%^66^. The flow cytometry strategy presented can be used in the future to explore the function of these cells in this tissue. Studies in humans have shown that hematopoietic cells represent only 25–45% of total SVF cells as there are many non-hematopoietic cells such as endothelial cells, pericytes, as well as other stromal cells^13^. Similarly, we observed here that CD45^+^ cells represent in median values approximately 35% and 51% of total SVF cells in MAT and SAT, respectively. In the CD45 negative population, we verified the presence of a population that although negative for common leukocyte markers, such as CD45, MHC-II and CD11b, binds to the antibody clone CH138A. Previous reports had shown that only neutrophils from bovine blood and milk and neutrophils and monocytes from water buffalo^67^ stained with this antibody. We show here that this antibody can also bind to non-leukocytes cells, as confirmed by absence of CD45 expression in the sorted CH138A^+^CD11b^−^MHC-II^−^CD45^−^ cell population. To the best of our knowledge the specificity of the CH138A mAb, to which these cells strongly bind, is unknown and it would be interestingly in the future to determine its specificity as it could greatly assist in better characterizing this cell population. Mesenchymal stromal cells were already shown to be present in bovine adipose tissue^16,17^. These cells are CD45^−^, CD11b^−^ and MHC-class II^−17^ and could therefore be one of the populations present in the CH138A^+^CD11b^−^MHC-II^−^CD45^−^ population. As another possibility, these cells may represent preadipocytes as these are negative to CD45 and CD11b expression and well known to be present in the adipose tissue^19^. Moreover, the sorted CH138A^+^CD11b^−^MHC-II^−^CD45^−^ cells have a similar morphology to the preadipocyte cellular line 3T3-L1^68^.

The frequency of cells in adipose tissue can be affected by several factors; one of those can be previous exposure to infection^53,69,70^. When we separate the data accordingly to previous exposure to *N. caninum*, as assessed by serology, an increased frequency in macrophages was observed in SAT of animals seropositive to *N. caninum*. These may suggest that in bovines, as already described in the murine model^53^, a previous infection challenge can affect the frequency of cellular populations in adipose tissue. On the other hand, animals seropositive to *N. caninum* showed lower frequencies of the CH138A^+^CD11b^−^MHC-II^−^CD45^−^ population in MAT and SAT, which further hints for an influence of infection in the bovine adipose tissue cell composition. Another factor that may have influenced the frequency of cell populations in bovine adipose tissue is the age of the animals. Nevertheless, in studies done in the murine model, aging does not appear to associate with major changes in the number of macrophages and eosinophils in adipose tissue^71^. As samples were randomly recovered from animals slaughtered for human consumption and not for research purposes, no information on the gestational/reproductive status, and lactational stage of the animals could be obtained. The influence that these parameters may have in the frequency of cells in adipose tissue is therefore unknown.

The present report, by providing a more thorough approach to characterize immune cell populations present in the bovine adipose tissue, and the blood as well, may thus be helpful for future studies addressing the immune response in those tissues. Bovine can also be an additional model to the widely used murine one in studies addressing adipose tissue immunobiology.

## Methods

### Animals

Samples were recovered from 27 female Holstein-Friesian cattle (*Bos taurus*) at a local slaughterhouse (14 animals for flow cytometry analysis and 13 for cell sorting experiments and morphological analysis). Information on the age of all animals included in this study is provided in Supplementary Tables 1 and 2. No animals were sacrificed for research purpose, since all tissue samples were randomly recovered from animals slaughtered for human consumption. Authorization to use animal’s by-products was given by the competent national authority Direção Geral de Alimentação e Veterinária (reference 0421/000/000/2015) and Institutional Ethics Committee at ICBAS.

### Sample collection

Sample collection was done as previously described^20^. Briefly, samples of peripheral blood, mesenteric adipose tissue (MAT) and subcutaneous adipose tissue (SAT) were collected right after slaughter. Blood samples were collected from the jugular vein into tubes with ethylene diamine tetracetate (EDTA, BD Vacutainer^®^). SAT (removed from the flank region) and MAT (collected from the fat surrounding the mesenteric lymph nodes, avoiding the lymph nodes) samples were placed in Dulbecco’s Modified Eagle Medium (DMEM) supplemented with 100 units/mL penicillin, 100 μg/mL streptomycin, 250 ng/mL amphotericin B and 10 mM HEPES buffer (all from Sigma-Aldrich, St Louis, USA) and transported immediately to the laboratory for further analysis in a container with a water bath at 38–39 °C.

### Isolation of peripheral blood leukocytes

Peripheral blood leukocytes (PBL) were isolated by a previously described methodology^20^. Briefly, whole blood was incubated with red blood lysis buffer solution [162,64 mM NH_4_Cl (Sigma-Aldrich), 9.98 mM Tris base (Merck), pH = 7,2]. For flow cytometry experiments, cells were passed through a 100-μm cell strainer, washed and resuspended in Dulbecco’s PBS, supplemented with 2% FBS (Gibco, MA, USA), 2 mM EDTA and 10 mM HEPES (all from Sigma-Aldrich) after centrifugation at 300 × g for 5 min at 4 °C. For cell sorting experiments, cells were passed through a 100-μm cell strainer, washed and resuspended in RPMI-1640 medium, supplemented with 10% FBS (Biowest, Nuaillé, France), 85 units/mL penicillin, 85 μg/mL streptomycin, 62.5 ng/mL of amphotericin B, 10 mM HEPES and 0.05 mM 2-mercaptoethanol (all from Sigma-Aldrich) (complete RPMI medium).

### Isolation of stromal vascular fraction cells

Stromal vascular fraction (SVF) cells were isolated by a previously described methodology^20^. Briefly, small pieces of adipose tissue (1–2 g of SAT or MAT) (avoiding blood vessels) were added to tubes containing Hanks’ balanced salt solution supplemented with 4% BSA, 10 mM HEPES and Liberase™ TL Research Grade (Roche Diagnostics, Risch-Rotkreuz, Switzerland) and incubated in a water bath up to 60 min at 37 °C, with manual shaking each 10 min. After water bath incubation, digested samples were homogenized to single-cell suspensions, passed through a 100-μm cell strainer (BD Biosciences Pharmingen, San Diego, CA) and centrifuged at 280 × g for 10 min at 4 °C. Cells at the bottom, corresponding to the SVF were resuspended in Dulbecco’s PBS, supplemented with 2% FBS (Biowest, Nuaillé, France), 2 mM EDTA and 10 mM HEPES (all from Sigma-Aldrich) for flow cytometry studies and complete RPMI medium for cell sorting experiments.

### Antibodies

To construct a seven-colour panel, antibodies were first labelled with conjugation kits, accordingly to manufacturer instructions, as commercially available antibodies for bovines are conjugated to a very limited number of fluorochromes. Namely, mouse anti-bovine CD11b (clone CC126, Bio-Rad) was conjugated to Phycoerythrin (PE) with PE conjugation kit (PE) (AbD Serotec), mouse anti-bovine CD14 (Clone CC-G33, Bio-Rad) was conjugated to peridinin-chlorophyll protein-cychrome 5.5 (PerCP-Cy5.5) with PerCP-Cy5.5 conjugation kit (PerCP-Cy5.5) (Bio-Rad), mouse anti-bovine MHC-II (clone CAT82A, Washington State University) was conjugated to PE-cychrome 7 (PE-Cy7) with PE-Cy7^®^ conjugation kit (Abcam, Cambridge, UK), mouse anti-bovine CD11c (clone BAQ153A, Washington S. U.) was conjugated to Allophycocyanin (APC) with APC conjugation kit (Abcam) and mAb clone CH138A (Washington State University) described to bind to granulocytes, was conjugated to allophycocyanin Cyanine 7 (APC-Cy7) with APC-Cy7^®^ conjugation kit (Abcam). Fluorescein isothiocyanate (FITC) anti-bovine CD45 (Clone CC1, Bio-Rad, Kidlington, UK) was commercially available. All the antibodies used in this study were titrated with the optimal concentration determined for adipose tissue samples.

### Flow cytometry analysis

Flow cytometry analysis was done as previously described, with slight modifications^20^. Dead cells were excluded from our analysis by including in our panel a fixable viability dye (FVD). For that, all samples, except single-stained and FVD-Fluorescence minus one (FMO) control, were first incubated with eFluor^®^ 506 Fixable Viability Dye (eBioscience, San Diego, CA) diluted 1: 1000 in Dulbecco’s PBS for 30 min at 4 °C. Cells were then washed 2 times with PBS. Before surface staining, cells were incubated with 100 μg/mL of purified bovine IgG (Sigma-Aldrich) in Dulbecco’s PBS, 2% FBS, 2 mM EDTA, 10 mM HEPES as a blocking reagent for elimination of nonspecific binding, similarly to what is done for human studies^72^. SVF cells were then surface stained for 30 min with the above-described monoclonal antibodies. Briefly, FITC anti-bovine CD45, PE anti-bovine CD11b, PerCP-Cy5.5 anti-bovine CD14, PE-Cy7 anti-bovine MHC-II, APC anti-bovine CD11c and mAb clone CH138A bound to APC-Cy7. Following primary antibody incubation, cells were washed, fixed with 2% formaldehyde, washed and resuspended in Dulbecco’s PBS, 2% FBS, 2 mM EDTA, 10 mM HEPES. In each experiment for gating selection, FMO controls were made for all markers used using at least one adipose tissue sample and PBL. Whenever possible, FMO controls were made with one sample of MAT and one sample of SAT. Isotype controls were not used for gate setting as they have been shown to be highly unreliable^72^. 5 × 10^5^ to 1 × 10^6^ total cells were stained. Data acquisition was performed in a FACSCanto™ II system (BD Biosciences, San Jose, CA) using the FACSDIVA™ software (BD) and compensated and analysed in FlowJo version 9.9.6. (FlowJo LLC, Ashland, OR). Beads (antibodies) or cell (FVD) staining were used for compensation. As recommended in flow cytometry analysis^73^, a dot plot with the time parameter was included to allow exclusion of event burst that could have occurred during acquisition. Also, a gate was drawn to allowed exclusion of aggregates/doublets and another to exclude cellular debris. Selection of live cells was made in the graphic of FVD versus CD11b, as this was the combination providing the best separation of cells without excluding cells with high autofluorescence. Exclusion of dead cells from the analysis was crucial as the frequency of live cells in all cells excluding cell debris and singlets, ranged between 30 to 70% in MAT and 14% to 70% in SAT. The results are presented as the frequency of cells within live cells or frequency within each cellular population. A biexponential transformation was applied to improve data visualization.

### Fluorescence-activated cell sorting

Due to the impossibility of performing the cell sorting in the same day of cell isolation, isolated SVF cells or PBL were incubated approximately 12 h in a Thermo Scientific Nunc UpCell 12 MultiDish in complete RPMI medium. After this incubation the plate was incubated 30 minutes at room temperature, after which cells were recovered, washed and resuspended in Dulbecco’s PBS, 2% FBS (Gibco, MA, USA), 5 mM EDTA and 25 mM HEPES (all from Sigma-Aldrich). Before surface staining, cells were incubated with 100 μg/mL of purified bovine IgG (Sigma-Aldrich) as a blocking reagent for elimination of nonspecific binding, similarly to what is done for human studies^72^. SVF cells were then surface stained for 30 min with the above-described monoclonal antibodies. Briefly, FITC anti-bovine CD45, PE anti-bovine CD11b, PerCP-Cy5.5 anti-bovine CD14, PE-Cy7 anti-bovine MHC-II, APC anti-bovine CD11c and mAb clone CH138A bound to APC-Cy7. In each experiment for gating selection, FMO controls were made for all markers and for all adipose tissue samples and PBL. Beads staining was used for automatic compensation. Data acquisition and cell sorting was done in a BD FACSAriaII and data analysis for gate section was done with the BD FACSDiva Software 8.01. A nozzle of 100 μm was used and the purity mode for the sorting was selected. Cells were collected in FACS tubes containing complete RPMI medium. Illustrative examples of gate selection for the cell sorting experiments are shown in Supplementary Figs. S3 and S6. This pseudocolor plots were obtained in FlowJo version 9.9.6.

### May-Grünwald-Giemsa staining

Cytospins of the abovementioned SVF cells and sorted cellular populations were prepared by cytocentrifugation at 1000 rpm in a Shandon Cytospin 3 centrifuge for 5 min. The slides were then methanol fixed, stained with May-Grünwald stain for 15 min followed by 30 min in 5% Giemsa stain (all from Merck, Darmstadt, Germany. Slides were then washed with distilled water, dried and mounted with Entellan^®^ (Merck).

### Immunocytochemistry

Cytospins of SVF cells isolated from MAT and SAT as well as leukocytes isolated from whole blood were prepared as described above and stained by a previously described protocol with some modifications^12^. Briefly, peroxidase activity was blocked by treatment with 3% hydrogen peroxide in methanol (Merck, Darmstadt, Germany) for 20 min. Slides were then incubated in a moist chamber for 20 min with normal rabbit serum (Dako, Glostrup, Denmark) diluted 1:5 in 10% BSA (Sigma), to eliminate non-specific staining. Excess serum was removed and the slides were incubated in a moist chamber overnight at 4 °C, with the antibodies described in detail above) (mouse anti-bovine CD14, CD11b, MHC-II, CD11c, CD45, and mouse mAb clone CH138A). Slides incubated with primary antibodies were then washed and incubated for 30 min at room temperature with the polyclonal rabbit anti-mouse biotinylated secondary antibody (Dako) diluted 1:200 and then with the avidin–biotin peroxidase complex (Dako), for a further 30 min. The colour in all slides was developed by incubation with 3,3′-diaminobenzidine (Dako). After counterstaining sections with Mayer’s Haematoxylin (Merck), slides were mounted in Entellan (Merck). A positive reaction was indicated by the presence of brown cytoplasmic staining.

### Detection of *N. caninum*-specific antibodies by ELISA

Bovine serum was screened for antibodies specific to *N. caninum* with a commercially available multi-species indirect ELISA kit (ID Screen^®^ *Neospora caninum* Indirect Multi-species, ID.Vet, Grabels, France) accordingly to manufacturer’s instructions. The following formula was applied to the obtained optical density (OD): [(OD sample-OD negative control)/(OD positive control-OD negative control)] × 100. Samples were considered negative if the result of this formula was lower than 40% and positive if equal or above 50% as recommended by the manufacturer. Animals with negative and positive samples were classified as seronegative and seropositive to *N. caninum*, respectively. We chose this kit to assess seropositivity to *N. caninum* as others have shown that this was one of the best-adjusted ELISA kit for the serological diagnosis of bovine neosporosis from the ones commercially available^74^.

### RNA isolation and real time PCR analysis

Total RNA extraction and cDNA synthesis were performed by a protocol similar to the one previously described in the murine model^53^. Briefly, RNA was extracted from total SVF cells (6 × 10^4^ cells), total PBL (6 × 10^4^ cells) and from sorted SSC-A^high^CD11b^−/+^CD14^−^MHC-II^−^CH138A^−^CD45^+^ cells (mast cells; 4 × 10^3^−1 × 10^4^ sorted cells), isolated as described above, using TriReagent™ (Sigma-Aldrich) according to the manufacturer’s instructions. All RNA samples were recovered in 10 μl of nuclease-free H_2_O and quantified using Nanodrop ND-1000 apparatus (Thermo Scientific). Synthesis of cDNA was then performed with 180–370 ng RNA (SVF cells), 70–380 ng RNA (PBL) and 70–300 ng RNA (sorted mast cells), prepared as described above in a 10 μl final volume using a Maxima^®^ First-Strand cDNA Synthesis kit for RT-quantitative PCR (Fermentas, Thermo Scientific), according to the manufacturer’s instructions. The PCR programme run (25 °C for 10 min; 50 °C for 30 min; 85 °C for 5 min) was performed in a TProfessional Basic Thermocycler (Biometra GmbH, Goettingen, Germany). The mRNA expression levels of protein tyrosine phosphatase receptor type C (*PTPRC*) and tryptase beta 2 (*TPSB2*) were determined by real-time PCR, using the Kapa SYBR Fast qPCR Kit (Kapa Biosystems Inc, Wilmington, MA) in a Rotor-Gene 6000 (Corbett life science, Sydney, Australia). As reference genes we used emerin (*EMD*) and MARVEL domain containing 1 (*MARVELD1*), as they were described to be stable for bovine adipose tissue^75^. The reaction was performed in a final volume of 10 μL containing 0.2 μM of each specific primer^75^: *MARVELD1* forward: GGCCAGCTGTAAGATCATCACA, *MARVELD1* reverse: TCTGATCACAGACAGAGCACCAT; *EMD* forward: GCCCTCAGCTTCACTCTCAGA, *EMD* reverse: GAGGCGTTCCCGATCCTT (all from Tib Molbiol, Berlin, Germany), *TPSB2* forward: ACCTGCCAAGATGCTCCAT, *TPSB2* reverse: GCTGGACATGACAAGAGATGC (Sigma), *PTPRC* forward: GGAAATCGCCCCTGTCTGATA, *PTPRC* reverse: TCGTGGTCTGCTCATCTTGA and 1 × Master Mix plus 1 μL of the newly-synthesized cDNA previously diluted 1:10. The PCR program run was as follows: 1) denaturation at 95 °C, 5 min; 2) amplification in 50 cycles (95 °C, 10 seconds; 62 °C, 20 seconds). We analysed real-time PCR data by the comparative threshold cycle (C_T_) method^76^. Individual relative gene expression values were calculated using the following formula: 2 ^−^^(C^T ^gene of interest − C^T ^constitutive gene)^ ^76^. *TPSB2* primers were designed with primer-BLAST tool^77^. For *TPSB2 gene*, we additionally sequenced the obtained PCR products to confirm their specificity (Supplementary Fig. S9c).

### Statistical analysis

Statistical significance of results was determined by non-parametric Wilcoxon matched-pairs signed rank test when two paired groups were analysed, non-parametric Friedman test with Dunn’s multiple comparisons test when three paired groups were analysed and non-parametric Mann-Whitney U test when two unpaired groups were analyse, calculated with GraphPad Prism 8.0 software. (*P ≤ 0.05; **P ≤ 0.01; ***P ≤ 0.001; ****P ≤ 0.0001). As the number of samples per groups is low (n = 14) and a normal distribution was not observed for all our data (normality tested with Shapiro-Wilk test and Kolmogorov-Smirnov test calculated with GraphPad Prism 8.0 software), non-parametric tests were used^78,79^.

The data presented in flow cytometry analysis is from 5 pooled independent experiments, with n = 2–3 animals/experiment, as indicated in respective figure legends. The data presented in morphological analysis represent medians of 7 animals per group pooled from 6 independent experiments.

## Supplementary information

Supplementary Information.

## Data Availability

The datasets generated during and/or analysed during the current study are available from the corresponding author on reasonable request.
