# Supplementary material for: Characterization of Myeloid Cellular Populations in Mesenteric and Subcutaneous Adipose Tissue of Holstein-Friesian Cows
Source: Sci Rep. 2020 Feb 4;10:1771. doi: 10.1038/s41598-020-58678-0 (PMC7000716; doi:10.1038/s41598-020-58678-0)
Supplement: Supplementary file 1 — Supplementary Information. [file 41598_2020_58678_MOESM1_ESM.pdf]

Characterization of myeloid cellular populations in mesenteric and subcutaneous adipose tissue of Holstein-Friesian cows

Bárbara M. Oliveira[1,2], Ana Pinto[1,2], Alexandra Correia[3,4], Paula G. Ferreira[1,2], Manuel Vilanova[1,3,4], Luzia Teixeira[1,2,*]

[1]ICBAS – Instituto de Ciências Biomédicas Abel Salazar, Universidade do Porto, Rua de Jorge Viterbo Ferreira, 228, 4050-313, Porto, Portugal.

[2]UMIB – Unidade Multidisciplinar de Investigação Biomédica, Universidade do Porto, Rua de Jorge Viterbo Ferreira, 228, 4050-313, Porto, Portugal.

[3]I3S – Instituto de Investigação e Inovação em Saúde, Universidade do Porto, Rua Alfredo Allen, 208, 4200-135 Porto, Portugal;

[4]IBMC – Instituto de Biologia Molecular e Celular, Rua Alfredo Allen, 208, 4200-135 Porto, Portugal.

*Address Correspondence to:

Luzia Teixeira, PhD

ICBAS - Instituto de Ciências Biomédicas Abel Salazar da Universidade do Porto. Rua de Jorge Viterbo Ferreira n.º 228, 4050-313 Porto, Portugal.

Telephone: (+351) 220428109; E-mail: lmteixeira@icbas.up.pt

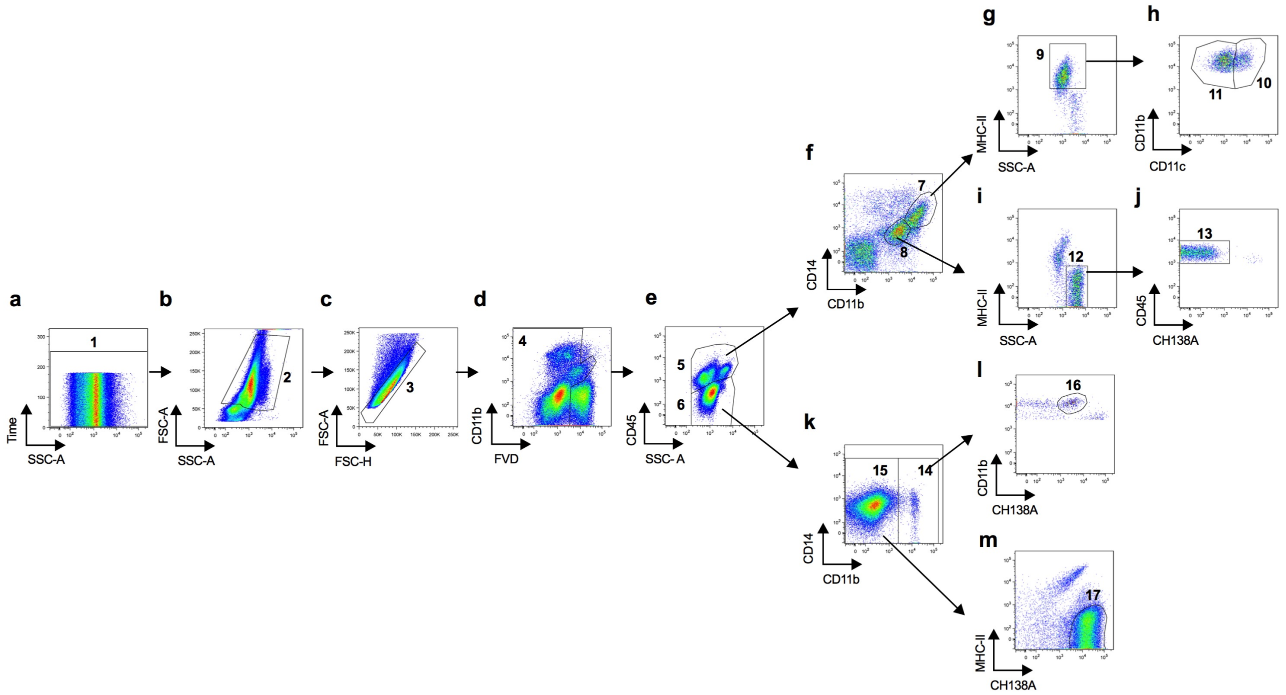

Supplementary Figure S1. Flow cytometry gating strategy used to define cell populations from the stromal vascular fraction (SVF) of bovine mesenteric adipose tissue (MAT). a) Time parameter allowed exclusion of events bursts (1), **b)** Selection of cells without debris (2) and **c)** singlets (3). **d)** Dead cells exclusion with Fixable Viability Dye (FVD) (4). **e)** Selection of CD45$^+$ (5) and CD45$^-$ (6) cells. **f)** Selection of CD14$^+$CD11b$^+$ (7) or CD11b$^{-/+}$CD14$^-$ cells (8). **g)** Gate used to analyse CD14$^+$CD11b$^+$MHC-II$^+$CD45$^+$ cells (macrophages) (9). **h)** Gate used to identify CD11c$^+$ (10) and CD11c$^-$ (11) macrophages. **i)** Selection of SSC-A^{high}MHC-II$^-$ cells (12). **j)** Gate used to analyse SSC-A^{high}CD11b$^{-/+}$CD14$^-$MHC-II$^-$CH138A$^-$CD45$^+$ cells (mast cells) (13). **k)** Selection of CD11b$^+$ (14) or CD11b$^-$ (15) cells in CD45$^-$ cells. **l)** Gate used to analyse CH138A$^+$CD11b$^+$ cells (neutrophils) (16). **m)** Gate used to analyse CH138A$^+$CD11b$^-$MHC-II$^-$CD45$^-$ cells (17). Pseudocolor plots are representative examples of analysed SVF cells isolated from MAT.

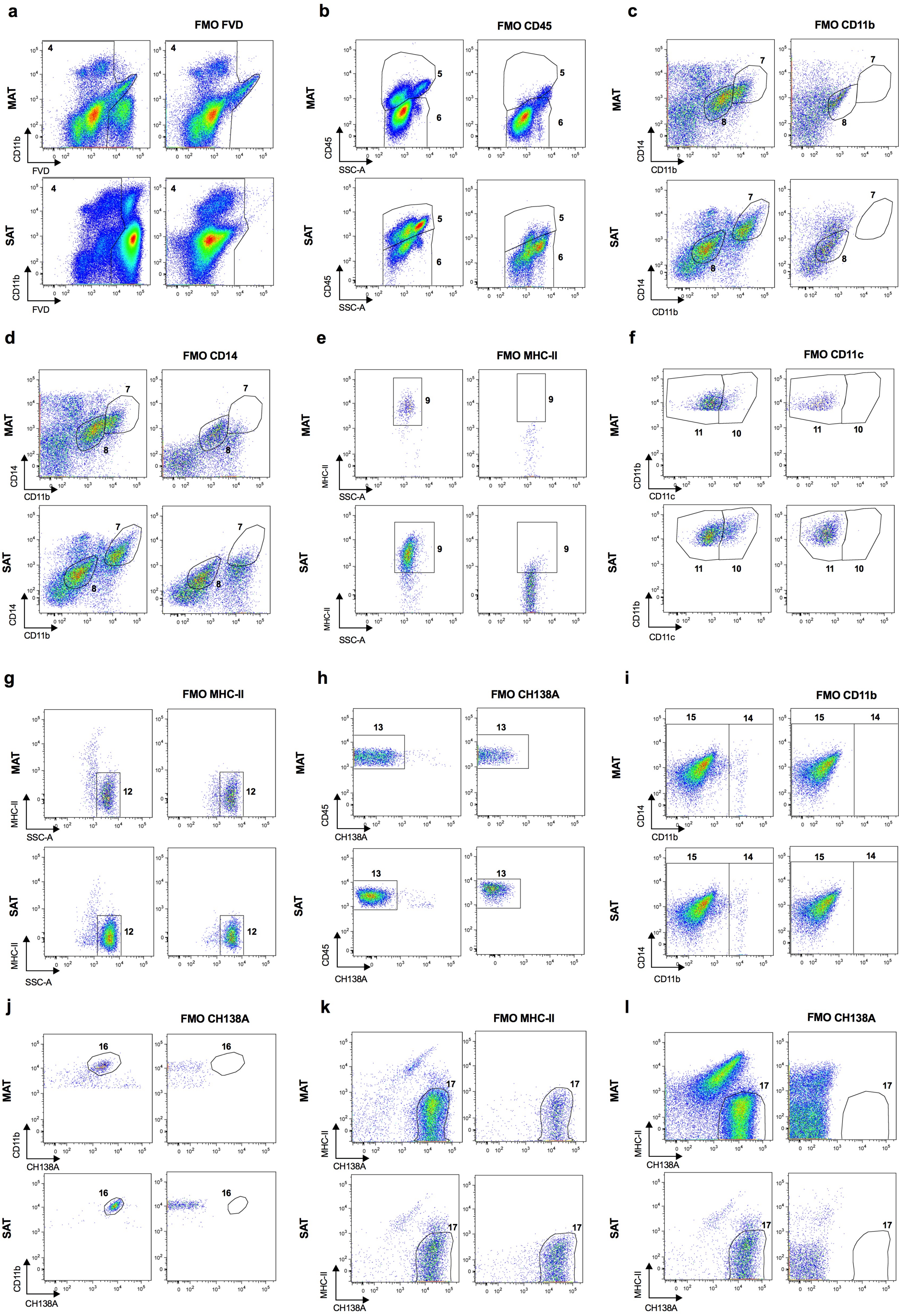
a
FMO FVD
MAT
SAT
CD11b
FVD
b
FMO CD45
CD45
SSC-A
c
FMO CD11b
CD14
CD11b
d
FMO CD14
CD14
CD11b
e
FMO MHC-II
MHC-II
SSC-A
f
FMO CD11c
CD11b
CD11c
g
FMO MHC-II
MHC-II
SSC-A
h
FMO CH138A
CD45
CH138A
i
FMO CD11b
CD14
CD11b
j
FMO CH138A
CD11b
CH138A
k
FMO MHC-II
MHC-II
CH138A
l
FMO CH138A
MHC-II
CH138A

Supplementary Figure S2. Fluorescence Minus One controls for gating strategy. Representative pseudocolor plots of stained cells and respective Fluorescence Minus One (FMO) controls used to determine correct gating for myeloid cell populations. **a)** Gate used to select FVD$^-$ cells (4) (left plot) and respective FMO (right plot), **b)** gate used to select CD45$^+$ cells (5) (left plot) and respective FMO (right plot), **c)** gate used to select CD11b$^+$ (7) and CD11b$^{-/+}$ (8) in CD45$^+$cells and respective FMO (right plot), **d)** gate used to selected CD14$^+$ cells (7) in CD45$^+$cells and respective FMO (right plot), **e)** gate used to select MHC-II$^+$ cells (9) in CD14$^+$CD11b$^+$CD45$^+$ cells (left plot) and respective FMO (right plot), **f)** gate used to select CD11c$^+$ cells (10) in CD14$^+$CD11b$^+$MHC-II$^+$CD45$^+$cells (left plot) and respective FMO (right plot), **g)** gate used to select MHC-II$^-$ cells (12) in SSC-A^{high}CD11b$^{-/+}$CD14$^-$CD45$^+$cells (left plot) and respective FMO (right plot), **h)** gate used to select CH138A$^-$ cells (13) in SSC-A^{high}CD11b$^{-/+}$CD14$^-$MHC-II$^-$CD45$^+$cells and respective FMO (right plot), **i)** gate used to select CD11b$^+$ (14) and CD11b$^-$ (15) in CD45$^-$ cells (left plot) and respective FMO (right plot), **j)** gate used to select CH138A$^+$ (16) in CD11b$^+$CD45$^-$ cells (left plot) and respective FMO (right plot), **k)** gate used to select MHC-II$^-$ (17) in CD11b$^-$CD45$^-$ cells (left plot) and respective FMO (right plot), **l)** gate used to select CH138A$^+$ (17) in CD11b$^-$CD45$^-$ cells (left plot) and respective FMO (right plot) in bovine mesenteric adipose tissue (MAT) and subcutaneous adipose tissue (SAT). Upon analysis of FMO controls it was observed that some populations in bovine adipose tissue had very high autofluorescence, clearly noticeable for the violet and blue laser. FMO control for FVD was crucial to avoid exclusion of the population with high autofluorescence from the analysis.

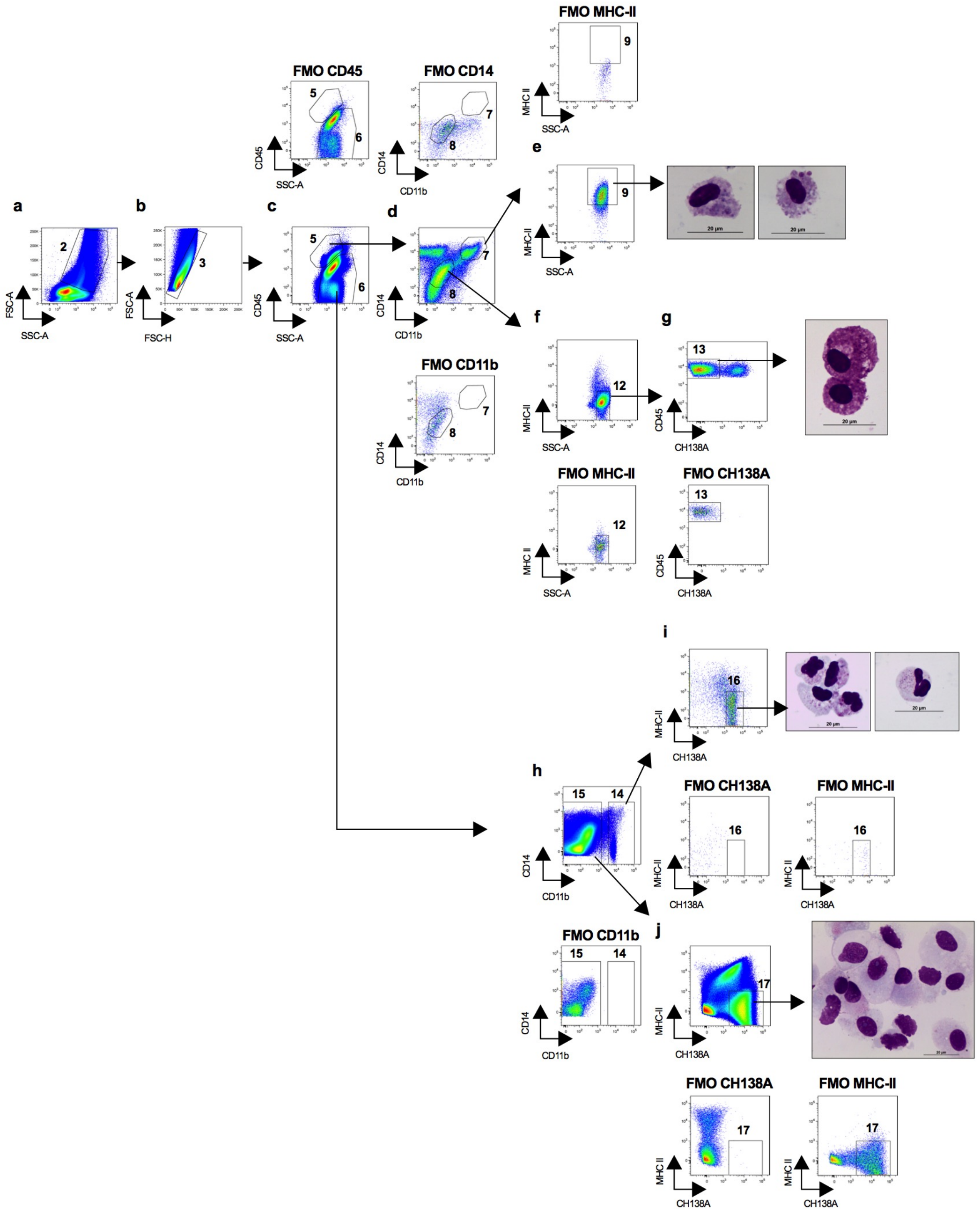

Supplementary Figure S3. Flow cytometry gating strategy used to define and sort cell populations from the stromal vascular fraction (SVF) of bovine subcutaneous adipose tissue (SAT). a) Selection of cells without debris (2) and **b)** singlets (3). **c)** Selection of CD45^{+} (5) and CD45^{-} cells (6). **d)** Selection of CD14^{+}CD11b^{+} (7) or CD11b$^{-/+}$CD14^{-} cells (8). **e)** Gate used to sort CD14^{+}CD11b^{+}MHC-II^{+}CD45^{+} cells (macrophages) (9). **f)** Selection of SSC-A^{high}MHC-II^{-} cells (12). **g)** Gate used to sort SSC-A^{high}CD11b$^{-/+}$CD14^{-}MHC-II^{-}CH138A^{-}CD45^{+} cells (mast cells) (13). **h)** Selection of CD11b^{+} (14) or CD11b^{-} (15) in CD45^{-} cells. **i)** Gate used to sort CH138A^{+}CD11b^{+} cells (neutrophils) (16). **j)** Gate used to sort CH138A^{+}CD11b^{-}MHC-II^{-}CD45^{-} cells (17). Respective Fluorescence Minus One (FMO) controls are presented. May-Grünwald-Giemsa staining of sorted populations are shown. Bar=20 µm in all micrographs. This is a representative example of gating strategy from 6 animals, from 5 independent experiments for SAT (Animal 15 to 20 in supplementary table 2).

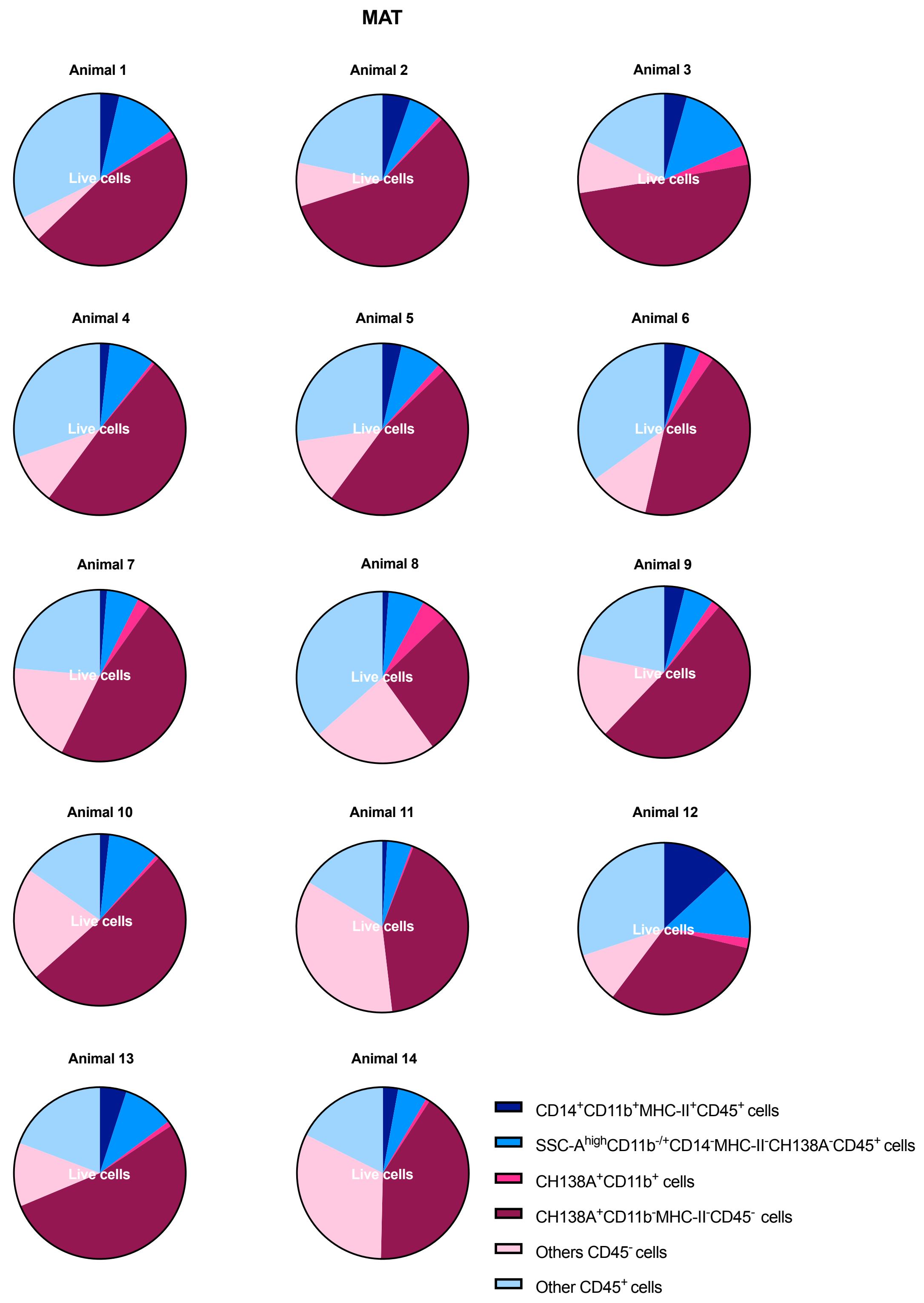

Supplementary Figure S4. Individual frequencies of myeloid cells in bovine mesenteric adipose tissue (MAT). Individual pie charts showing the relative frequency of CD14^{+}CD11b^{+}MHC-II^{+}CD45^{+} cells (macrophages), SSC-A^{high}CD11b$^{-/+}$CD14^{-}MHC-II^{-}CH138A^{-}CD45^{+} cells (mast cells), CH138A^{+}CD11b^{+} cells (neutrophils), CH138A^{+}CD11b^{-}MHC-II^{-}CD45^{-} cells, other CD45^{-} and CD45^{+} cells on total live cells, as indicated, in MAT from each animal included in the study.

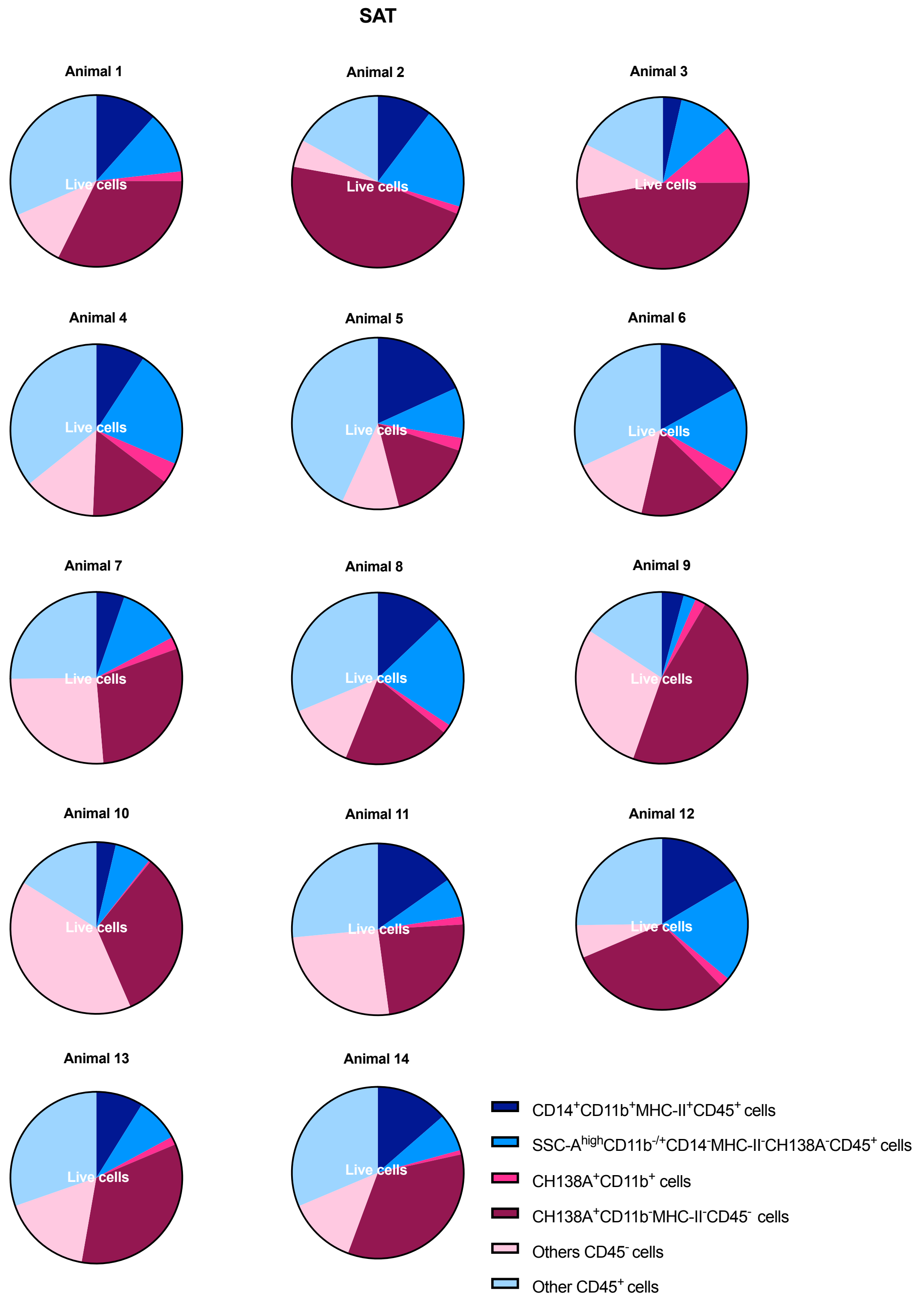

Supplementary Figure S5. Individual frequencies of myeloid cells in bovine subcutaneous adipose tissue (SAT). Individual pie charts showing the relative frequency of $CD14^+CD11b^+$MHC-II^+CD45^+ cells (macrophages), SSC-$A^{high}CD11b^{-/+}CD14^-$MHC-$II^-CH138A^-CD45^+$ cells (mast cells), $CH138A^+CD11b^+$ cells (neutrophils), $CH138A^+CD11b^-$MHC-II^-CD45^- cells, other $CD45^-$ and $CD45^+$ cells on total live cells, as indicated, in SAT from each animal included in the study.

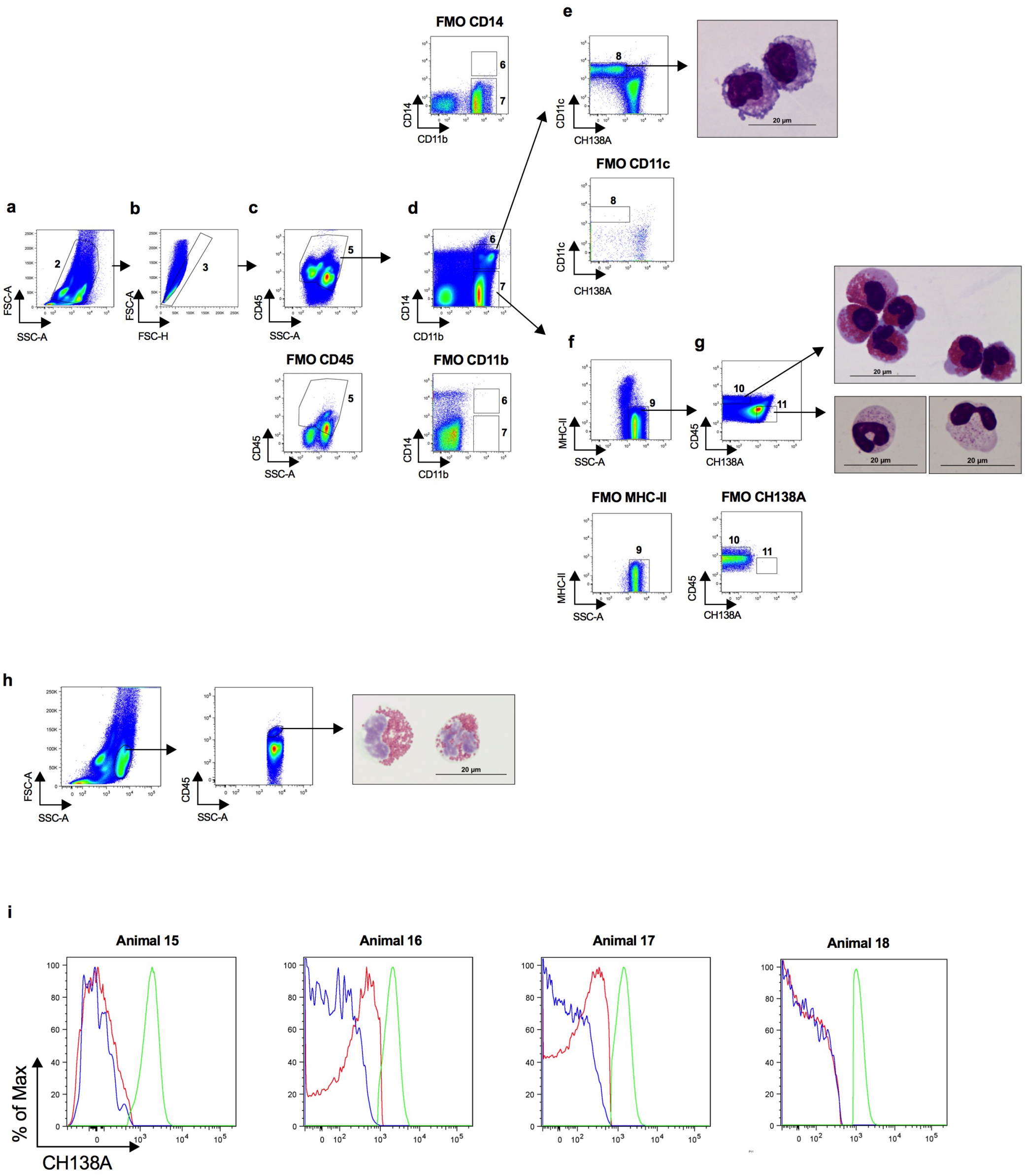

Supplementary Figure S6. Flow cytometry gating strategy used to define and sort cell populations from bovine peripheral blood leukocytes (PBL). **a)** Selection of cells without debris (2) and **b)** singlets (3). **c)** Selection of CD45^{+} cells (5). **d)** Selection of CD14^{+}CD11b^{+} (6) or CD11b^{+}CD14^{-} cells (7). **e)** Gate used to sort CD14^{+}CD11b^{+}CD11c^{+} cells (monocytes) (8). **f)** Selection of SSC-A^{high} MHC-II^{-} cells (9). **g)** Gate used to sort SSC-A^{high}CD11b^{+}CD14^{-}MHC-II^{-} CH138A$^{-/int}$ cells (10) and CH138A^{+}CD11b^{+}CD14^{-}MHC-II^{-}SSC-A^{high} cells (11) populations (eosinophils and neutrophils, respectively). Respective Fluorescence Minus One (FMO) controls are presented. This is a representative example of gating strategy from 4 animals from 4 independent experiments. **h)** Gate used to sort SSC-A^{high} autofluorescence^{high} cells from an unstained PBL sample. May-Grünwald-Giemsa staining of sorted populations is shown. Bar=20 µm in all micrographs. **i)** Illustrative example of histograms for CH138A expression in eosinophils (red line) and neutrophils (green line) in different animals from four distinct cell-sorting experiments. Similar histograms were obtained for flow cytometry experiments. The negative control (FMO for CH138A) is also shown for the gate corresponding to eosinophils (blue line).

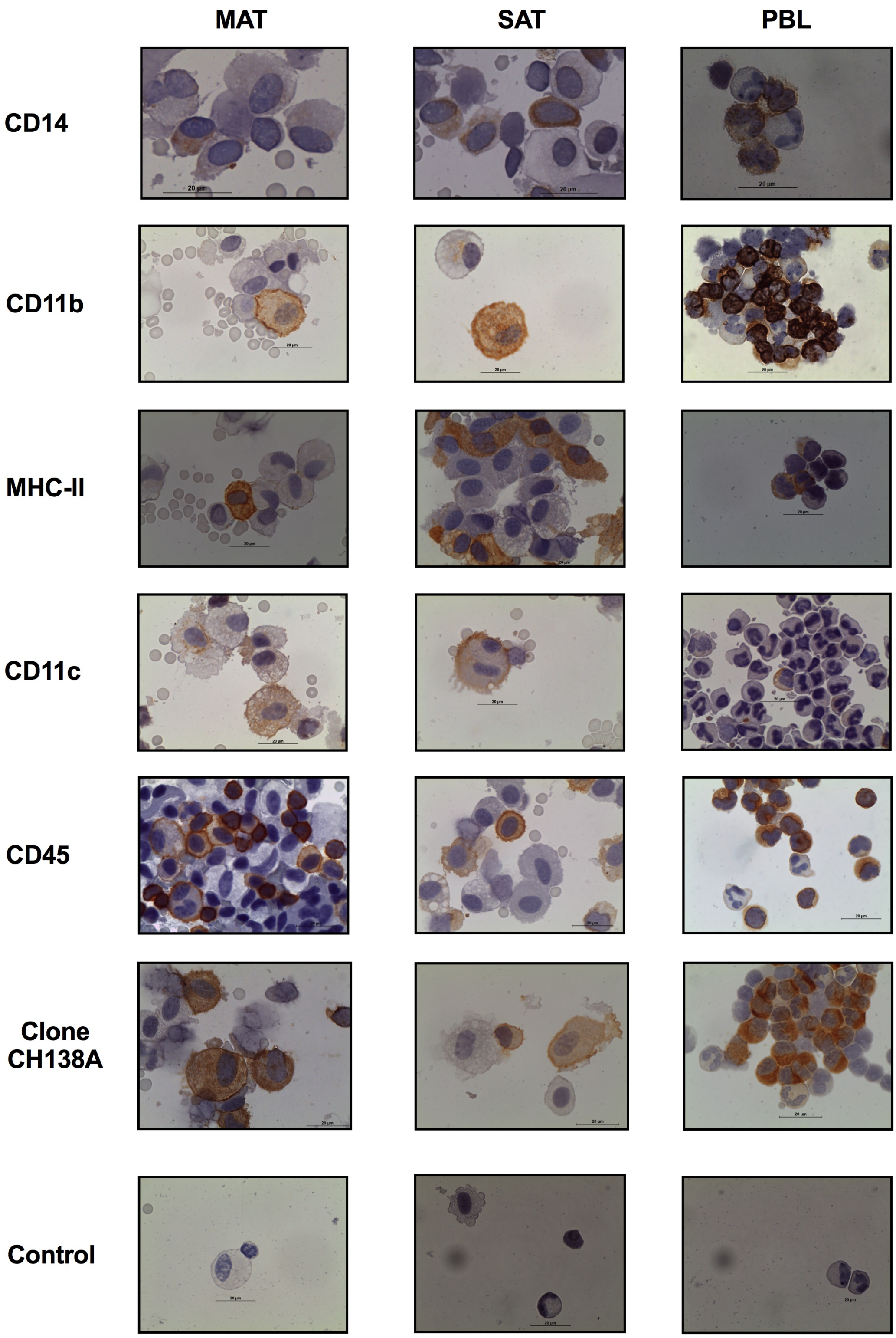

Supplementary Figure S7. Immunocytochemistry analysis of CD14, CD11b, MHC-II, CD11c, CD45 and mAb clone CH138A in the stromal vascular fraction (SVF) cells isolated from mesenteric and subcutaneous adipose tissue (MAT and SAT, respectively) and in peripheral blood leukocytes (PBL), recovered from bovines as indicated. Cells were specifically stained (brown coloration) with a monoclonal mouse anti-bovine CD14, CD11b, MHC-II, CD11c and CD45 and counterstained with haematoxylin. The monoclonal antibody from clone CH138A, whose specificity is unknown, was also tested. Control (cells without addition of any antibody). Bar=20μm in CD14, CD11b, MHC-II, CD11c, control and clone CH138A and Bar=50 μm in CD45. These are representative images from three independent experiments with n= 2-3 for MAT, SAT and PBL.

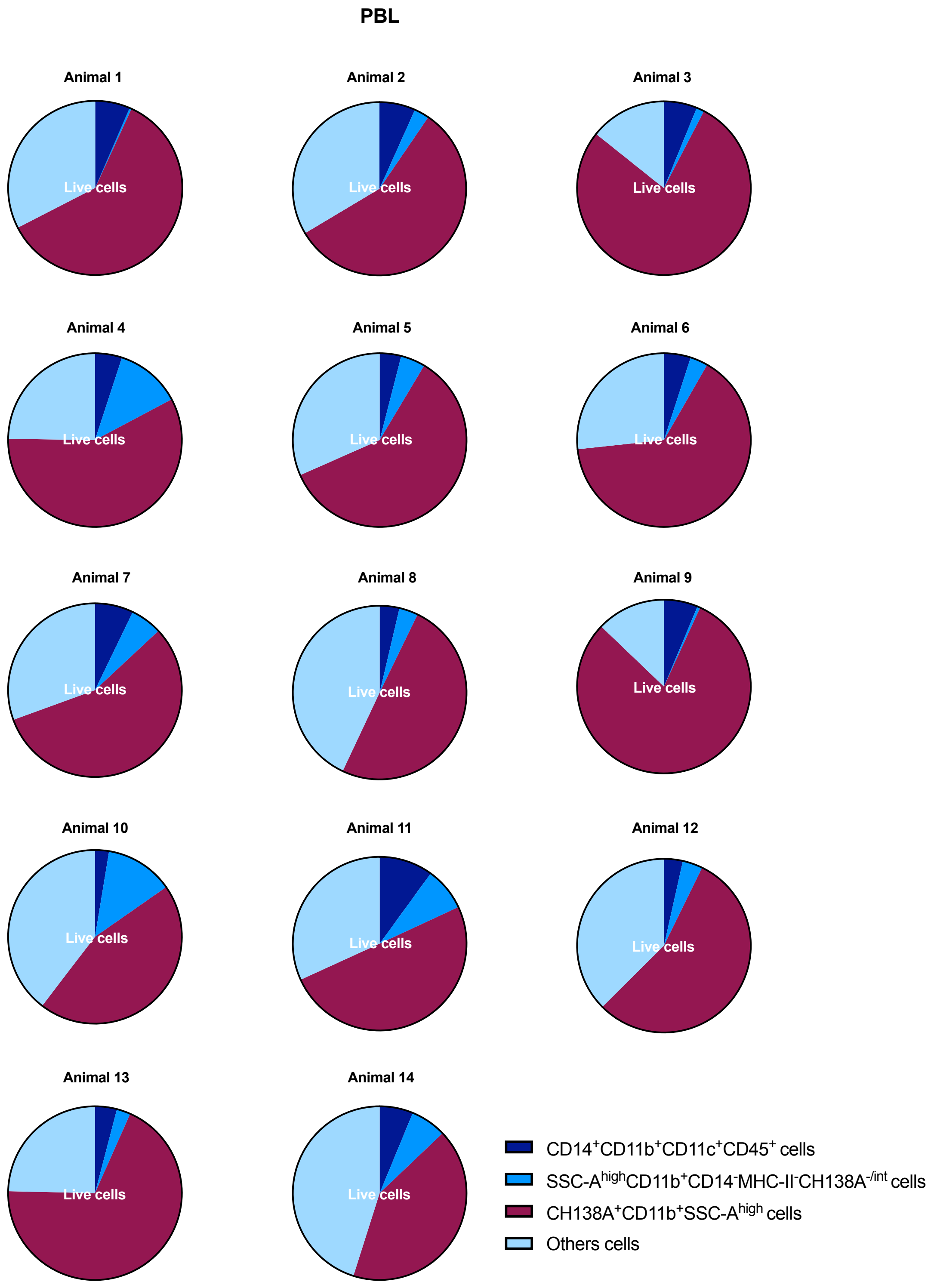

Supplementary Figure S8. Individual frequencies of myeloid cells in bovine peripheral blood leukocytes (PBL). Individual pie charts showing the relative frequency of $CD14^{+}CD11b^{+}CD11c^{+}$ cells (monocytes), $SSC\text{-}A^{high}CD11b^{+}CD14^{-}MHC\text{-}II^{-}CH138A^{-/int}$ cells (eosinophils) and $CH138A^{+}CD11b^{+}SSC\text{-}A^{high}$ cells (neutrophils) and other cells on total live cells in PBL from each animal included in the study.

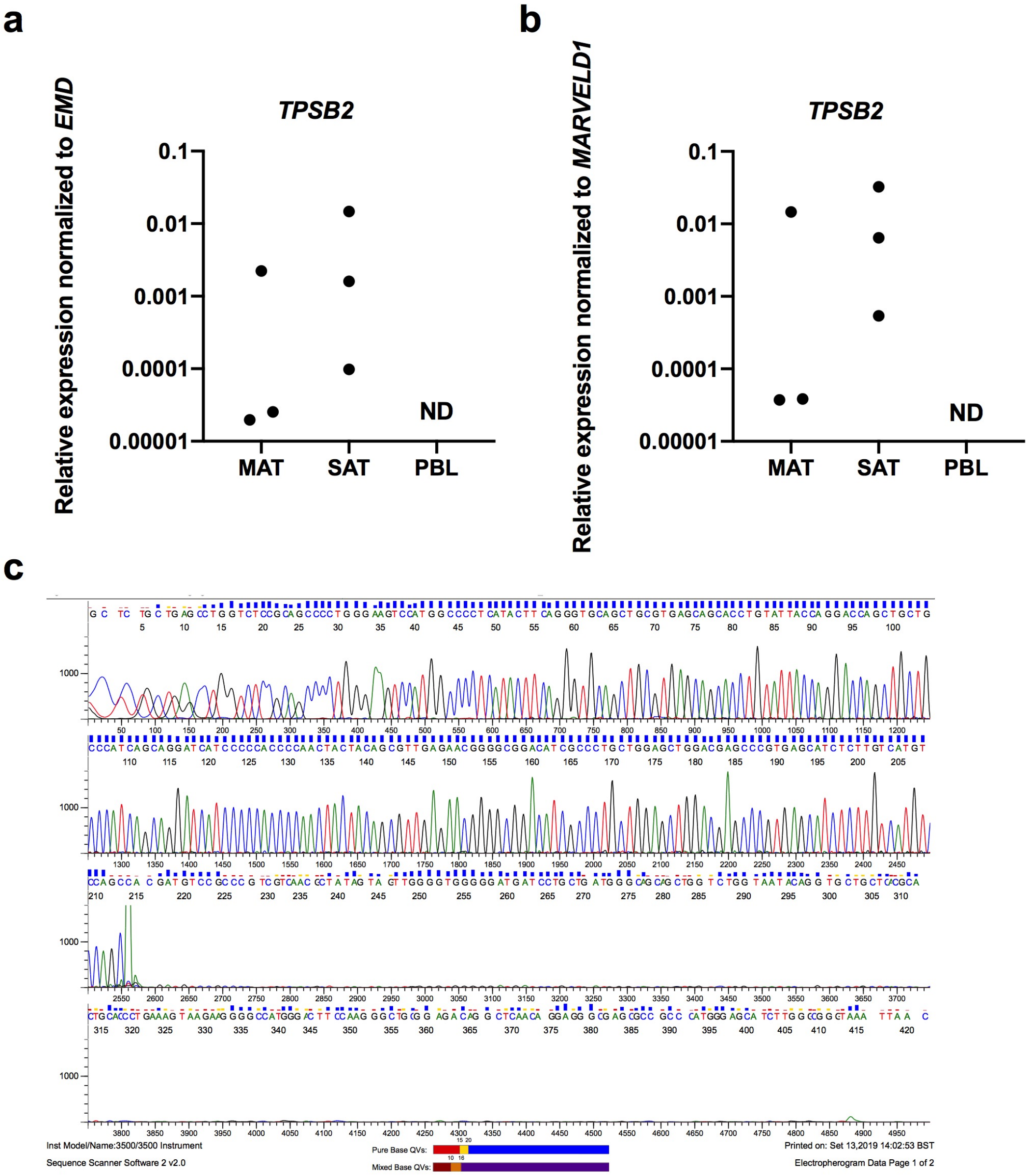

Supplementary Figure S9. Tryptase beta 2 expression in mast cells population. Relative levels of tryptase beta 2 (*TPSB2*) mRNA normalized to **a)** emerin (*EMD*) and **b)** MARVEL domain containing 1 (*MARVELD1*) mRNA as indicated, detected by real-time PCR in sorted SSC-A^{high}CD11b$^{-/+}$CD14^{-}MHC-II^{-}CH138A^{-}CD45^{+} cells (mast cells) isolated from the stromal vascular fraction (SVF) of mesenteric and subcutaneous adipose tissue (MAT and SAT, respectively) and in peripheral blood leukocytes (PBL). Each symbol represents an individual animal (n=3 for MAT, SAT and PBL). ND= not detected for *TPSB2* mRNA expression (signal detected for both constitutive genes *EMD* and *MARVELD1* mRNA). **c)** Representative result of electropherogram resulting from the sequencing of the PCR products obtained in a). Example shown for mast cells sorted from SAT from animal 16. Percent Identity of 100% with *Bos taurus* tryptase beta 2 (*TPSB2*) mRNA were obtained when the sequences were compared against the NCBI database using BLAST algorithm. Similar results were obtained for the other animals. DNA sequencing of PCR fragments was performed at the Genomics i3S Scientific Platform. Both PCR and sequencing products were purified using illustra™ Sephadex™ G-50 Fine DNA Grade according to the manufacturer's protocol. Sequencing products were analysed by capillary electrophoresis on a 3500 Genetic Analyzer (Applied Biosystems). Electropherograms were visualized using Sequence Scanner Software 2 v2.0 (Applied Biosystems).

a

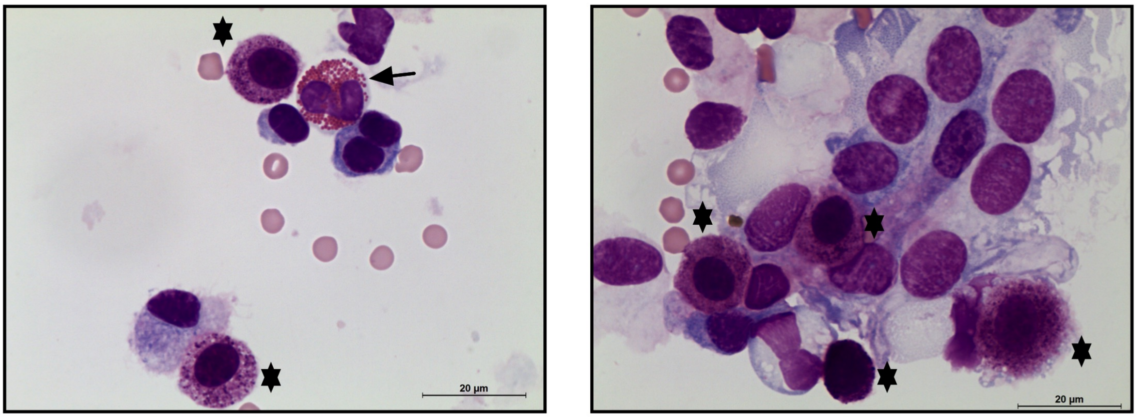

b

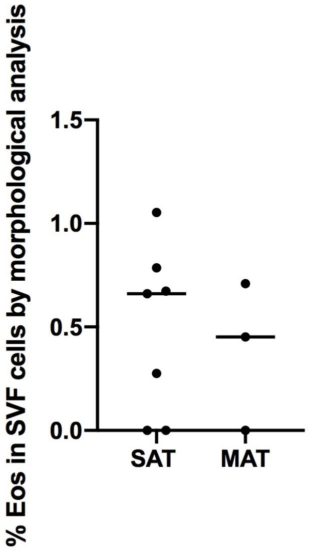

c

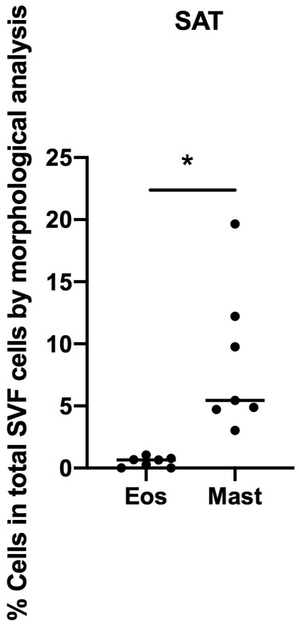

Supplementary Figure S10. Morphological quantification of eosinophils in bovine adipose tissue. a) Illustrative examples of May-Grünwald-Giemsa staining of stromal vascular fraction (SVF) cells isolated from bovine subcutaneous adipose tissue (SAT). Representative eosinophils (arrow) and mast cells (star) are shown. Bar=20 µm in all micrographs. **b)** Frequency of eosinophils (Eos) in the SVF of SAT and mesenteric adipose tissue (MAT) evaluated by morphological analysis. Each symbol represents an individual animal. Bars represent medians of 7 animals pooled from 6 independent experiments in SAT and 3 animals pooled from 3 independent experiments in MAT. **c)** Frequency of eosinophils (Eos) and mast cells (Mast) in the SVF of SAT evaluated by morphological analysis. Each symbol represents an individual animal. Bars represent medians of 7 animals per group pooled from 6 independent experiments. Statistically significant differences between different tissues are indicated. (Wilcoxon matched-pairs signed rank test; *, $P<0.05$).

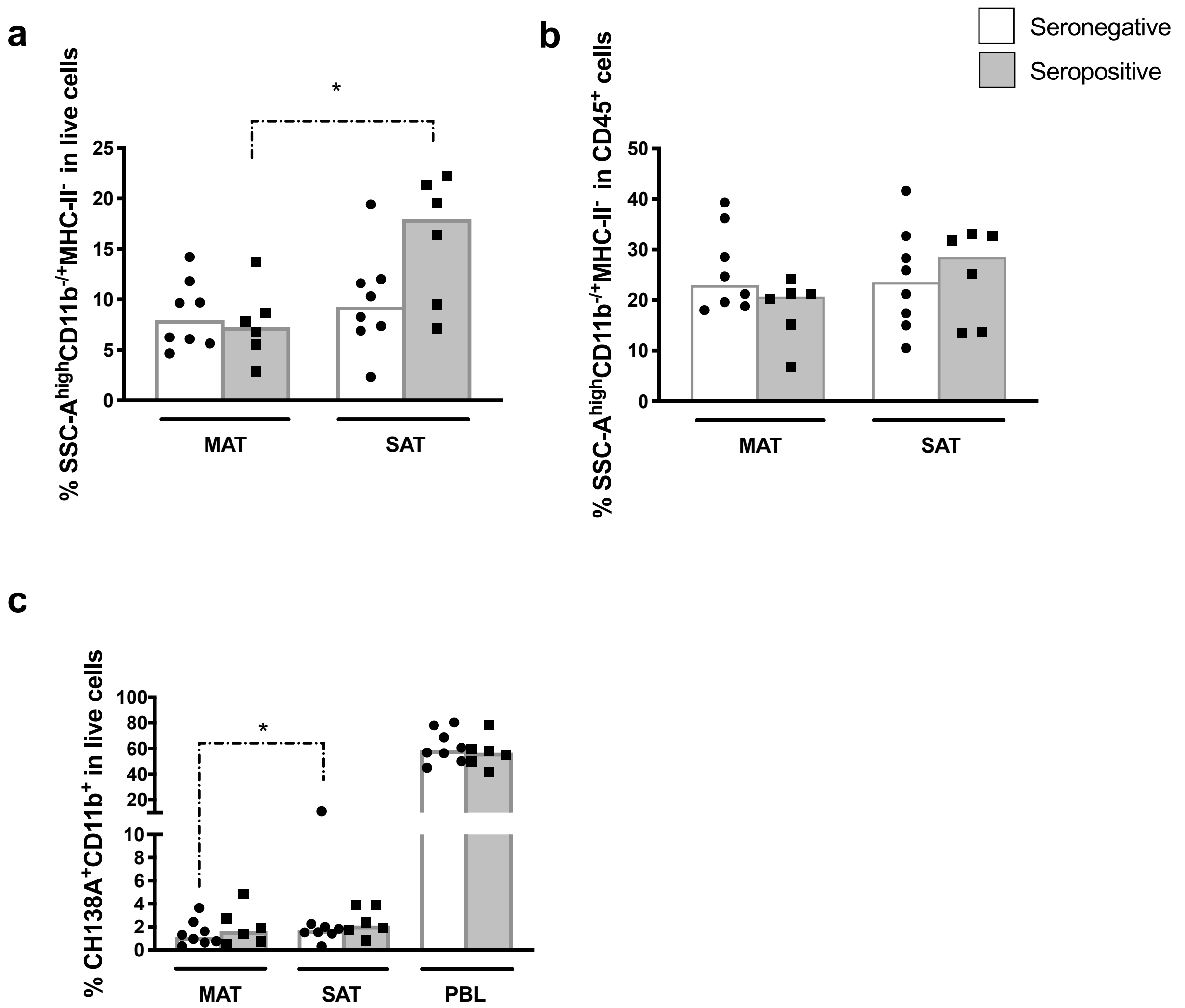

Supplementary Figure S11. Granulocytes in *N. caninum* seropositive animals. Frequencies of SSC-A^{high}CD11$b^{-/+}$CD14^{-}MHC-II^{-}CH138A^{-}CD45^{+} cells (mast cells) in **a)** total live stromal vascular fraction cells (SVF) and **b)** CD45^{+} cells isolated from mesenteric and subcutaneous bovine adipose tissue (MAT and SAT, respectively) from bovines seronegative (white bars) or seropositive (grey bars) to *N. caninum* as indicated. **c)** Frequencies of CH138A^{+}CD11b^{+} cells (neutrophils) in total SVF cells isolated MAT and SAT and in leukocytes isolated from whole blood (PBL) from bovines seronegative (white bars) or seropositive (grey bars) to *N. caninum*. Each symbol represents an individual animal. Bars represent means of 6 or 8 bovines per group pooled from 5 independent experiments. Statistically significant differences between different tissues are indicated. (Mann-Whitney U, *, P<0.05).

Supplementary Table S1. Age of animals included in the study of flow cytometric analysis in months.

Animal number	Age (months)
**1**	8
**2**	12
**3**	66
**4**	101
**5**	49
**6**	97
**7**	25
**8**	48
**9**	95
**10**	52
**11**	25
**12**	39
**13**	58
**14**	21

Supplementary Table S2. Age of animals included in the study of fluorescence-activated cell sorting, *TPSB2* and *PTPRC* expression and eosinophil morphological analysis in months.

Animal number	Age (months)
**15**	28
**16**	79
**17**	57
**18**	16
**19**	59
**20**	61
**21**	26
**22**	34
**23**	23
**24**	47
**25**	17
**26**	55
**27**	59
